# Effects of water, sanitation, and hygiene interventions on detection of enteropathogens and host-specific faecal markers in the environment: a systematic review and individual participant data meta-analysis

**DOI:** 10.1016/S2542-5196(23)00028-1

**Published:** 2023-03-06

**Authors:** Andrew Mertens, Benjamin F Arnold, Jade Benjamin-Chung, Alexandria B Boehm, Joe Brown, Drew Capone, Thomas Clasen, Erica Fuhrmeister, Jessica A Grembi, David Holcomb, Jackie Knee, Laura H Kwong, Audrie Lin, Stephen P Luby, Rassul Nala, Kara Nelson, Sammy M Njenga, Clair Null, Amy J Pickering, Mahbubur Rahman, Heather E Reese, Lauren Steinbaum, Jill Stewart, Ruwan Thilakaratne, Oliver Cumming, John M Colford, Ayse Ercumen

**Affiliations:** a Division of Epidemiology and Biostatistics, University of California, Berkeley, CA, USA; b Division of Environmental Health Sciences, University of California, Berkeley, CA, USA; c Department of Civil and Environmental Engineering, College of Engineering, University of California, Berkeley, CA, USA; d Francis I Proctor Foundation and Department of Ophthalmology, University of California, San Francisco, CA, USA; e Department of Epidemiology and Population Health, Stanford University, Stanford, CA, USA; f Department of Civil and Environmental Engineering, Stanford University, Stanford, CA, USA; g Department of Medicine, Stanford University, Stanford, CA, USA; h Division of Infectious Diseases and Geographic Medicine, Stanford University, Stanford, CA, USA; i Department of Environmental Science and Engineering, Gillings School of Global Public Health, Michael Hooker Research Center, University of North Carolina, Chapel Hill, NC, USA; j Department of Environmental and Occupational Health, Indiana University Bloomington, Bloomington, IN, USA; k Department of Environmental Health, Rollins School of Public Health, Emory University, NE, Atlanta, GA, USA; l Department of Environmental and Occupational Health Sciences, University of Washington, Seattle, WA, USA; m Department of Disease Control, London School of Tropical Medicine & Hygiene, London, UK; n Department of Biobehavioral Health, Pennsylvania State University, PA, USA; o Ministério da Saúde, Instituto Nacional de Saúde Maputo, Maputo, Mozambique; p Kenya Medical Research Institute, Nairobi, Kenya; q Mathematica, Princeton, NJ, USA; r Environmental Interventions Unit, Infectious Diseases Division, Dhaka, Bangladesh; s Center for the Ecology of Infectious Diseases, University of Georgia, Athens, GA, USA; t Department of Forestry and Environmental Resources, North Carolina State University, Raleigh, NC, USA

## Abstract

**Background:**

Water, sanitation, and hygiene (WASH) improvements are promoted to reduce diarrhoea in low-income countries. However, trials from the past 5 years have found mixed effects of household-level and community-level WASH interventions on child health. Measuring pathogens and host-specific faecal markers in the environment can help investigate causal pathways between WASH and health by quantifying whether and by how much interventions reduce environmental exposure to enteric pathogens and faecal contamination from human and different animal sources. We aimed to assess the effects of WASH interventions on enteropathogens and microbial source tracking (MST) markers in environmental samples.

**Methods:**

We did a systematic review and individual participant data meta-analysis, which included searches from Jan 1, 2000, to Jan 5, 2023, from PubMed, Embase, CAB Direct Global Health, Agricultural and Environmental Science Database, Web of Science, and Scopus, of prospective studies with water, sanitation, or hygiene interventions and concurrent control group that measured pathogens or MST markers in environmental samples and measured child anthropometry, diarrhoea, or pathogen-specific infections. We used covariate-adjusted regression models with robust standard errors to estimate study-specific intervention effects and pooled effect estimates across studies using random-effects models.

**Findings:**

Few trials have measured the effect of sanitation interventions on pathogens and MST markers in the environment and they mostly focused on onsite sanitation. We extracted individual participant data on nine environmental assessments from five eligible trials. Environmental sampling included drinking water, hand rinses, soil, and flies. Interventions were consistently associated with reduced pathogen detection in the environment but effect estimates in most individual studies could not be distinguished from chance. Pooled across studies, we found a small reduction in the prevalence of any pathogen in any sample type (pooled prevalence ratio [PR] 0·94 [95% CI 0·90–0·99]). Interventions had no effect on the prevalence of MST markers from humans (pooled PR 1·00 [95% CI 0·88–1·13]) or animals (pooled PR 1·00 [95% CI 0·97–1·03]).

**Interpretation:**

The small effect of these sanitation interventions on pathogen detection and absence of effects on human or animal faecal markers are consistent with the small or null health effects previously reported in these trials. Our findings suggest that the basic sanitation interventions implemented in these studies did not contain human waste and did not adequately reduce exposure to enteropathogens in the environment.

**Funding:**

Bill and Melinda Gates Foundation and the UK Foreign and Commonwealth Development Office.

## Introduction

Every year, diarrhoea kills an estimated 525 000 children younger than 5 years.^1^ Enteropathogens (pathogens causing infections of the intestinal track) are transmitted from infected individuals' faeces to new hosts through a diverse set of interconnected environmental pathways, including contaminated water or food, hands, fomites, and vectors (eg, flies). Sources of faecal contamination include open defecation, unsafe sanitation facilities that do not isolate waste from the environment, and domestic animals. Water, sanitation, and hygiene (WASH) improvements have been promoted to reduce childhood enteric infections by reducing environmental person-to-person exposure to pathogens transmitted via the faecal–oral route. Traditionally, trials of WASH interventions have primarily focused on documenting health outcomes, such as caregiver-reported diarrhoea, without measuring intermediate outcomes along the causal chain, such as pathogens in environmental samples to characterise exposure. Such measurements can illuminate underlying mechanisms of interventions and offer explanations for intervention success or failure. Inspecting the causal chain is especially important given the small or null effects on child diarrhoea and growth in WASH trials.^2^, ^3^, ^4^, ^5^, ^6^

**Research in context**

**Evidence before this study**

Children in areas with poor drinking water, sanitation, and hygiene (WASH) conditions have increased diarrhoeal disease and reduced growth. Rigorous trials of WASH interventions from the past 5 years have shown mixed efficacy in reducing diarrhoeal disease in children and no improvements in child growth. Quantifying the effect of WASH improvements on enteric pathogens in environmental samples and on contamination originating from human versus animal sources might help elucidate if interventions successfully interrupt the causal pathway between poor WASH, environmental exposure to faecal pathogens, and child health. Most previous studies and meta-analyses on the effect of WASH interventions on faecal contamination in the environment have focused on faecal indicator bacteria (FIB). These studies have shown FIB reductions in water and on hands due to water treatment and handwashing, respectively, but no effects from sanitation. However, naturalised FIB can be present in the environment without faecal contamination, and poorly correlate with actual pathogen presence, affecting the interpretation of these findings. FIB also cannot distinguish between faecal contamination from humans and animals; this information can help identify whether the absence of health effects from sanitation interventions are due to zoonotic disease transmission from unsafely managed animal faeces. Applications of advanced analytic techniques to environmental sampling in low-income countries allow detection and enumeration of a range of enteropathogens, and faecal markers associated with specific hosts for microbial source tracking (MST). The evidence on WASH effects on pathogens and MST markers has not been previously synthesised. Therefore, we did a systematic review and individual participant data (IPD) meta-analysis of WASH intervention studies to assess if interventions reduced the prevalence and abundance of enteropathogens or MST markers in the domestic environment.

**Added value of this study**

We identified six eligible intervention studies that measured enteropathogens and MST markers in environmental samples and obtained data from five studies. Studies consistently indicated that the interventions were associated with reduced detection of pathogens, and some pathogens (eg, adenovirus and Shigella) showed large reductions, but most effect estimates in individual studies were null. The IPD meta-analysis approach allowed us to increase precision by pooling study-specific estimates to detect a small reduction in the prevalence of any type of pathogen in any type of sample. There was no overall intervention effect on the prevalence of human or animal MST markers. This study uses advanced methods to enumerate enteropathogens and host-specific faecal markers in a range of environmental samples, including understudied reservoirs such as soil. We provide the first synthesis of evidence of the effect of WASH interventions on these important targets to advance our understanding of the environmental mechanisms of interventions beyond the available evidence on effects on FIB.

**Implications of all the available evidence**

The environmental sampling in the studies in our review was mostly focused on onsite sanitation interventions that provided or promoted improved latrines with pits or septic tanks at the household or community level. The small reduction in pathogen prevalence in the environment, when pooled across all studies, might explain the small effect these interventions had on child health in the parent studies. Taken together, this evidence suggests that the sanitation interventions implemented in the studies in our review achieved a small reduction in faecal contamination in the environment. More comprehensive WASH interventions, such as safely managed water and sanitation, including safe disposal or treatment of excreta from both humans and animals, are potentially needed to reduce environmental contamination sufficiently to improve child health. We note that only a small number of intervention studies measured our targets of interest, and we identified no studies that assessed the effect of water treatment, hygiene, or safely managed or sewered sanitation interventions on pathogens and MST markers in the domestic environment, except for one study on a combined household water treatment and hygiene intervention in which data were not available for IPD analysis. Also, pathogen targets and analytic methods varied by study, reducing comparability. Future research would benefit from environmental sampling following implementation of a more diverse and comprehensive set of WASH interventions. Such studies should enumerate a common range of pathogen targets and use standardised laboratory methods for a given target and environmental matrix.

To date, WASH intervention studies measuring environmental contamination have mostly relied on faecal indicator bacteria (FIB), such as *Escherichia coli* as a proxy for a wide range of enteropathogens, including bacteria, viruses, protozoa, and helminths. Sampling has primarily focused on drinking water (and, to a smaller extent, hands and food) whereas other pathways, such as soil and surfaces, have received less attention.^7^ Household water treatment and handwashing have been associated with reduced FIB in drinking water^8^ and on hands,^9^, ^10^ respectively, whereas sanitation interventions have had little effect on FIB in drinking water or on hands, objects, surfaces, soil, and flies.^7^ However, FIB are imperfect predictors of faecal contamination, pathogen presence, and ultimate health risk. While *Escherichia coli* in drinking water is correlated with increased risk of diarrhoea,^11^ FIB can also originate from non-faecal sources^12^ and generally correlate poorly with pathogens in the environment.^13^ In addition, FIB are found in both human and animal faeces, and their detection in the environment cannot differentiate the source of contamination.^11^, ^12^, ^14^

Applications of advanced molecular methods to environmental sampling in low-income settings can offer advantages over FIB measurements in characterising environmental contamination. These methods can directly detect a range of enteropathogens,^15^, ^16^ and microbial source tracking (MST) methods aim to distinguish between human and animal faecal sources through detection of unique molecular characteristics of faecal microorganisms strongly associated with specific animal hosts.^17^ We assessed the effect of WASH interventions in low-income countries on enteropathogens and MST markers in the domestic environment with a systematic review and individual participant data (IPD) meta-analysis, which allows combining observation-level data from studies with standardised statistical methods.

## Methods

### Search strategy and selection criteria

**Figure 1:**
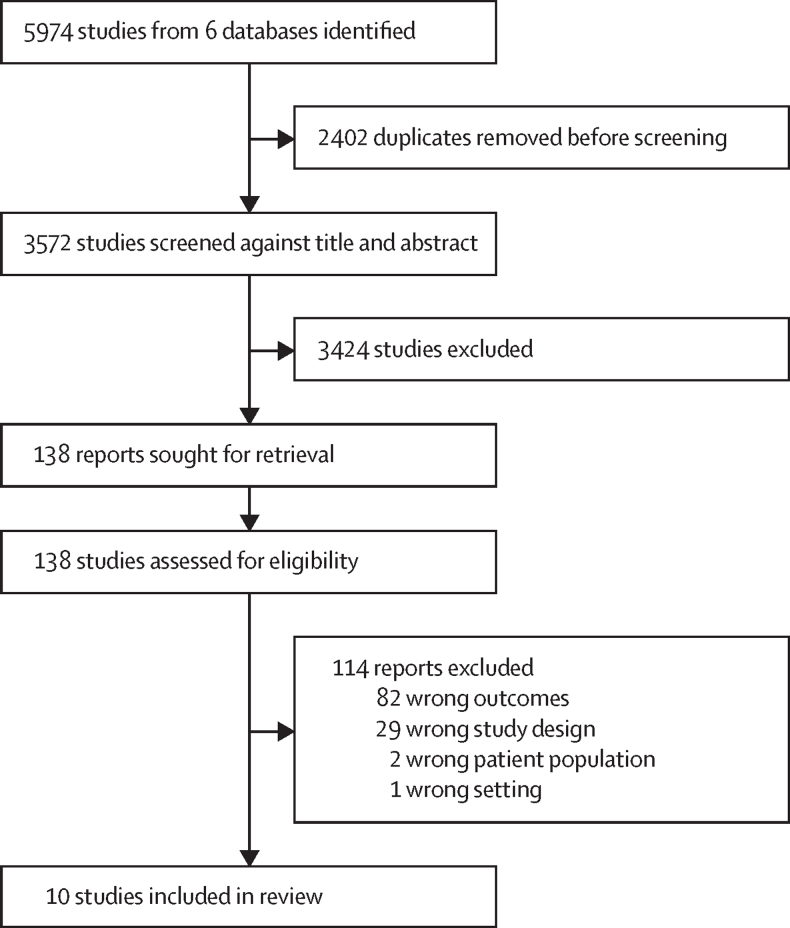
Study selection

We did a systematic review and individual participant data meta-analysis on the effects of WASH interventions on enteropathogens and MST markers in environmental samples. We searched PubMed, Embase, CAB Direct Global Health, Agricultural and Environmental Science Database, Web of Science, and Scopus (appendix pp 13–14). We included studies meeting the following inclusion criteria: (1) prospective studies with a water, sanitation, or hygiene intervention and concurrent control (ie, randomised controlled trial, matched cohort, controlled before-and-after study), consistent with previous WHO burden of disease reviews;^18^, ^19^ (2) measured pathogens or MST markers in environmental samples; and (3) measured child anthropometry, diarrhoea, or pathogen-specific infections. We restricted the search to studies measuring child health outcomes to estimate associations between environmental contamination and child health in a separate analysis.^20^ We included studies published from Jan 1, 2000, to Jan 5, 2023, to reflect recent advances in laboratory methods but we did not limit our search to any specific method (eg, molecular, culture-based, and microscopy). We excluded studies that only measured FIB. We limited our search to studies in English. Using Covidence systematic review software, one reviewer (AM) screened abstracts, and two independent reviewers (AM and RT) examined the full texts of short-listed articles with differences resolved with a third reviewer (AE). One reviewer (AM) forward and backward searched the citations of included articles (ie, checked the bibliographies of included studies and the studies that cited the included studies). We followed PRISMA reporting guidelines (figure 1, appendix pp 15–20). We used an adapted version of the Newcastle-Ottawa scale to evaluate bias (appendix pp 21–23).^21^

### Data collection and analysis

For each eligible study, we requested individual sample data from authors, and excluded studies in which data were not shared. Before sharing data, all personal identifiers such as GPS locations were removed, and indirect identifiers such as sampling dates were coarsened to a monthly resolution.

We did not prespecify specific enteropathogens or markers as outcomes because each study measured a different set of targets. We used the pathogens and MST markers measured in the included studies to generate two composite measures as our primary outcomes: (1) detection of any pathogenic target or (2) any MST target, in any sample type collected during the same sampling round from the same compound, where a compound was defined by the original studies as a set of households with common courtyards, water sources, or latrines. Because many targets were infrequently detected in individual studies, composite outcomes allowed us to pool information from studies that focused on different targets and sample types, leveraging the IPD approach for increased statistical precision. We also analysed the prevalence of any pathogen and any MST marker separately for each sample type (eg, water, hand rinses, soil, and flies). Secondary outcomes included the prevalence of specific pathogen class (any viruses, any bacteria, any protozoa, any helminths), the prevalence of MST markers from specific host types (human or animal), and the prevalence and abundance of individual enteropathogens and MST markers. We excluded general MST markers that are not host-specific from our analysis.

We compared outcomes between the intervention and control groups of each study. We estimated prevalence ratios (PRs) using modified Poisson regressions.^22^ For abundance outcomes, we used linear regressions to estimate differences in log_10_-transformed gene copies and negative binomial regressions to estimate ratios of soil-transmitted helminth (STH) egg counts. Because of repeated sampling or clustered designs in some studies, we used the Huber Sandwich Estimator to calculate robust standard errors using either the randomisation cluster or compound as the unit of independence.^23^ For abundance measures, we imputed values below the limit of detection (LOD) with half the LOD, and values below the limit of quantification (LOQ) with the midpoint between the LOD and LOQ using LOD and LOQ values supplied by data contributors. We limited our analysis of abundance measures to targets in which more than 50% of samples were within the range of quantification (ROQ).

Although estimated intervention effects from randomised trials should be unconfounded, covariate adjustment might increase statistical efficiency and improve exchangeability with matched cohorts and non-randomised trials.^24^ Therefore, we adjusted estimates for the treatment group and potential confounders chosen via a prescreening procedure. A prespecified set of potential confounding covariates (ie, potential predictors of each outcome) was prescreened using bivariate likelihood ratio tests, and those associated with the outcome with a p value less than 0·2 were included in the model for each outcome. As a sensitivity analysis, we also used LASSO penalised regressions to select adjustment covariates. We prescreened the following variables if they were measured within an included study: number of people in the household, age and education of primary caregiver, asset-based household wealth, number of rooms, construction materials (eg, walls, floor, and roof), access to electricity, land ownership, and if anyone in the household works in agriculture. These variables reflect sociodemographic conditions that are commonly considered potential confounders in WASH studies. When analysing binary outcomes, we only included one potential confounder per ten positive samples, or per ten negative samples if less than 50% of samples were negative, as per recommended best practices for the numbers of events per variable.^25^ We did not estimate prevalence ratios for targets with less than five positive or negative values for a given sample type. We did a complete case analysis for missing outcomes. For continuous covariates, we excluded any with more than 50% missingness, and for categorical covariates, we used a missing category. Given the heterogeneity across studies (eg, local WASH conditions, climate, urbanisation, population density, regional infectious disease patterns, and intervention type), we first individually estimated study-specific effects, and then pooled the effect estimates across studies using random-effects models, which allow for heterogeneity in intervention effects by assuming study-specific estimates come from a normal distribution of true effects.^25^ Estimates were pooled when outcome data were available for four or more studies and were fit using restricted-maximum likelihood with the metafor package (version 3.0-2) in R.^26^ We did not pool abundance estimates because of issues in standardising quantitative PCR (qPCR) methods across sites and the small number of available abundance estimates.^27^, ^28^

We did subgroup analyses by season (dry *vs* wet), animal ownership (at least one *vs* no animal owned) and pathogens (with *vs* without zoonotic transmission). The wet season for each study was defined as the 6 months of highest country-level average rainfall.^28^ Reported animal ownership was intended as an indicator for the potential presence of animal faecal contamination; we note that this is an imperfect proxy and there is no standardised metric for capturing the likelihood of animal faecal contamination in the domestic setting.^29^ The pathogens we considered as potentially zoonotic were *Campylobacter jejuni, Campylobacter coli, Salmonella, Yersinia enterocolitica, Clostridioides difficile, Cryptosporidium, Giardia*, and *Ascaris*.^30^ We classified *Ascaris* as potentially zoonotic because *Ascaris lumbricoides* and *Ascaris suum* cross-infect humans and pigs, and the microscopy methods used in the studies in our review do not distinguish between them.^30^, ^31^, ^32^ When studies detected virulence genes associated with specific *E coli* pathotypes (ie, enteroaggregative *E coli*, enteropathogenic and enterohemorrhagic *E coli*, Shiga toxin-producing *E coli*, enteroinvasive *E coli*, and enterotoxigenic *E coli*), we classified Shiga toxin-producing *E coli* and enteropathogenic *E coli* (due to atypical enteropathogenic *E coli*) as zoonotic.^30^ We used linear regression models estimating prevalence differences to assess additive interaction by examining the p values on the interaction terms between the treatment and the indicator variable for the subgroup; additive interaction has been argued to better capture public health importance than multiplicative interaction.^33^

We also assessed heterogeneity by study-level characteristics, including setting, study design, intervention uptake and time between intervention onset, and environmental sampling. There was little heterogeneity in urbanicity within any individual study. Therefore, we pooled estimates separately for rural versus urban studies. We also separately pooled estimates from randomised versus quasi-experimental studies, studies with high versus low intervention uptake and studies with shorter (≤1 year) versus longer (>1 year) follow-up between intervention onset and sampling. We compared pooled estimates between strata with Wald tests. Analyses were done in R version 4.0.4. Analysis scripts are publicly available (https://github.com/amertens/wash-ipd). Our systematic review search strategy and statistical analysis plan were preregistered and are available on Open Science Framework

### Role of the funding source

The funder of the study had no role in study design, data collection, data analysis, data interpretation, or writing of the report.

## Results

The systematic review was done on Jan 19, 2021, and returned 3572 results after removing duplicates. Of these, 3424 were excluded by abstract screening, and of 138 short-listed studies, ten were eligible after full-text screening. The ten articles reported environmental assessments from six unique intervention studies: the WASH Benefits Bangladesh (WBB)^3^ and WASH Benefits Kenya (WBK) trials,^4^ the MapSan study in Mozambique,^34^ the Gram Vikas study in India,^35^ the Total Sanitation Campaign (TSC) trial in India,^6^ and the CHoBI7 trial in Bangladesh^36^ (table 1). Data were obtained from all studies except CHoBI7 in which individual participant data were not shared; this trial was excluded from our analysis. For MapSan, additional data were shared from an analysis unpublished at the time of the search.^37^ For the TSC trial,^6^ only data on village-level source water quality were available. For the WBB and MapSan trials, multiple substudies within the trials collected samples from different subsets of participants at different times; therefore, we report the results of individual publications separately rather than combined by trial.

**Table 1:** Characteristics of included publications

	**Parent study**	**Study design**	**Intervention**	**Time between intervention and environmental sampling**	**Location**	**Sample types**	**Targets**	**Analytic method**	**Number of samples**
Capone et al (2022)^37^	..	..	..	About 2 years	..	Flies caught in latrine and kitchen	Panel of 16 enteric pathogens and MST markers	qPCR	86
Boehm et al (2016)^40^	WASH Benefits Bangladesh	Cluster-randomised trial	Latrine upgrades, child potties, and scoops for faeces disposal	4 months	Rural Bangladesh	Stored drinking water, child hands, and soil	Rotavirus, general, human, avian, and ruminant faecal markers	qPCR	1482
Holcomb et al (2021)^41^	MapSan	Controlled before and after study	Latrine upgrades	About 1 year	Urban Mozambique	Source and stored water, household and latrine soil, and food	General, human, and avian faecal MST markers	qPCR	353
Odagiri et al (2016)^42^	Total Sanitation Campaign	Cluster-randomised trial	Latrine upgrades	About 1 year	Rural India	Source water	*V cholerae*, rotavirus, and adenovirus, and general, human, and animal faecal markers	qPCR, microscopy	60
Reese et al (2017)^43^	Gram Vikas	Matched cohort study	Latrine upgrades and piped water	6–10 years	Rural India	Source and stored water	*Vibrio cholerae*, Shigella	Slide agglutination serotyping	3452
Kwong et al (2021)^44^	..	..	..	About 2 years	..	Courtyard soil	Soil-transmitted helminths	Microscopy	1396
Steinbaum et al (2019)^45^	WASH Benefits Kenya	Cluster-randomised trial	Latrine upgrades, child's potties, and scoops for faeces disposal	About 2 years	Rural Kenya	Courtyard soil	Soil-transmitted helminths	Microscopy	2149
Fuhrmeister et al (2020)^46^	..	..	..	16–35 months	..	Stored drinking water, children's and mothers' hands, and soil	Pathogenic *Escherichia coli*, norovirus, Giardia	qPCR	2601
Capone et al (2021)^47^	..	..	..	About 1 year	..	Household and latrine soil	Panel of 18 enteric pathogens	qPCR	88

qPCR=quantitative PCR. MST=microbial source tracking.

All studies assessed WASH intervention effects on diarrhoeal disease and growth in children younger than 5 years. Three studies were cluster-randomised controlled trials (WBB, WBK, and TSC). MapSan was a controlled before-and-after study with control and intervention sites matched on compound size and time of enrolment. Gram Vikas was a matched cohort study in which control and intervention villages were matched on 12 preintervention WASH and socioeconomic characteristics. With the Newcastle-Ottawa scale, studies had low risk of bias due to blinded outcome assessments, with the Gram Vikas and MapSan studies having a lower rating due to higher loss to follow-up and lack of randomisation (appendix pp 21–23). WBB, WBK, TSC, and Gram Vikas were done in rural settings, and MapSan in an urban setting. All included studies evaluated sanitation interventions (table 1). TSC and MapSan focused on sanitation alone. The WBB and WBK trials included individual and combined WASH and nutrition interventions but pathogens and MST markers in environmental samples were only measured in the sanitation and control groups. The Gram Vikas study evaluated a combined piped drinking water and sanitation intervention. The CHoBI7 trial (excluded because no data were shared) evaluated a combined water treatment and hygiene intervention. No included studies evaluated drinking water supply and treatment or hygiene interventions alone.

All sanitation interventions evaluated were onsite (ie, non-sewered) technologies delivered at the household or community level. None of the interventions met the Sustainable Development Goal standard of safely managed sanitation because no intervention included excreta treatment or offsite removal, and they would be classified as basic sanitation (or limited sanitation if participants shared latrines with neighbours, such as in the MapSan trial). The WASH Benefits studies provided new or upgraded improved latrines for each household in enrolled compounds, child potties and sani-scoops for faeces removal. In the WBB trial, latrines were dual-pit latrines with a water seal, and in WBK plastic latrine slabs were used to improve existing latrines. MapSan provided pour-flush latrines draining to septic tanks, shared by multiple households. TSC promoted construction of a pour-flush latrine with a single pit and Y-joint for a second pit, subsidised post hoc by government funding. In the Gram Vikas study, a non-governmental organisation provided materials for the construction of pour-flush latrines in each household in selected villages and built community water tanks and piped distribution systems providing household connections. When every household in the village completed latrine construction, the water system was turned on for the whole village.

Latrine access and use was higher in intervention households than control households in all studies. Definitions of latrine quality varied, including improved, clean, hygienic, or functional latrines, or latrines with a functional water seal, as observed by field staff. In four studies, 78–97% of intervention recipients had access to these types of facilities, compared with 18–45% of controls.^3^, ^4^, ^35^, ^38^ The TSC trial had the lowest effect on latrine access, with 38% of intervention compounds having functional latrines compared with 10% of controls.^6^ Latrine use in intervention households was variable and especially low among children, and safe management of child and animal faeces was uncommon. In WBB, 94% of adults were observed to defecate in a hygienic latrine in structured observations but only 54% of children were observed using the latrine or potty and 15% of animal faeces were observed to be removed with the sani-scoop.^39^ In WBK, reported safe disposal of child faeces dropped from 77% 1 year after intervention to 37% after 2 years.^4^ In TSC, 50% of households reported children using a latrine,^6^ and in Gram Vikas, 35% of intervention villages reported disposing of child faeces in improved latrines.^35^

Environmental samples were collected from 4 months^40^ to 6–10 years^35^ after intervention delivery, with most studies collecting samples 1–2 years after intervention (table 1). Sample types included source and stored drinking water, children's and mothers' hand rinses, soil from the courtyard, household and latrine areas, and flies caught in latrines and kitchens. Food samples were collected in one study^41^ but were not included in our analysis because only nine samples were positive for MST targets. The number of samples in individual studies varied from 60^42^ to 2107.^35^ Our pooled dataset included 12 184 samples, with 40 156 observations for pathogen or MST marker prevalence.

The studies measured a range of bacterial, viral, protozoan, and helminthic pathogens, including pathogenic *E coli, Vibrio cholerae, Shigella, C jejuni, C coli, Salmonella, Yersinia, C difficile*, rotavirus, norovirus, sapovirus, adenovirus, astrovirus, enterovirus, *Cryptosporidium, Giardia, Entamoeba histolytica, A lumbricoides*, and *Trichuris trichiura* (appendix pp 25–38). The MST markers included human (HumM2, HF183, BacHum, *Methanobrevibacter smithii*), animal (BacCan, BacCow), ruminant (BacR), and avian (GFD) faecal markers (appendix pp 25–38). Most studies used qPCR or RT-qPCR (table 1). One study used slide agglutination serotyping to detect *V cholerae* and *Shigella*.^43^ One study detected *Cryptosporidium* oocysts and *Giardia* cysts with direct fluorescent antibody microscopy.^42^ Two studies enumerated STH eggs by microscopy.^44^, ^45^

Many targets had low or no variation. Of 267 unique combinations of study, sample type, and target, 18 had no positive values, 41 had less than ten positive values, and two had less than ten negative values. Therefore, 206 of 267 sample-target combinations had sufficient variability to estimate a PR and be individually included in our IPD analysis. Among these, pathogen prevalence ranged from 7 (1·4%) of 496 for *Giardia* on mothers' hands^46^ to 886 (62·1%) of 1426) for *Ascaris* in soil,^44^ and the prevalence of MST markers ranged from 12 (2·4%) of 493) for HumM2 on children's hands^40^ to 356 (97·5%) of 365) for BacCow on mothers' hands.^46^

**Figure 2:**
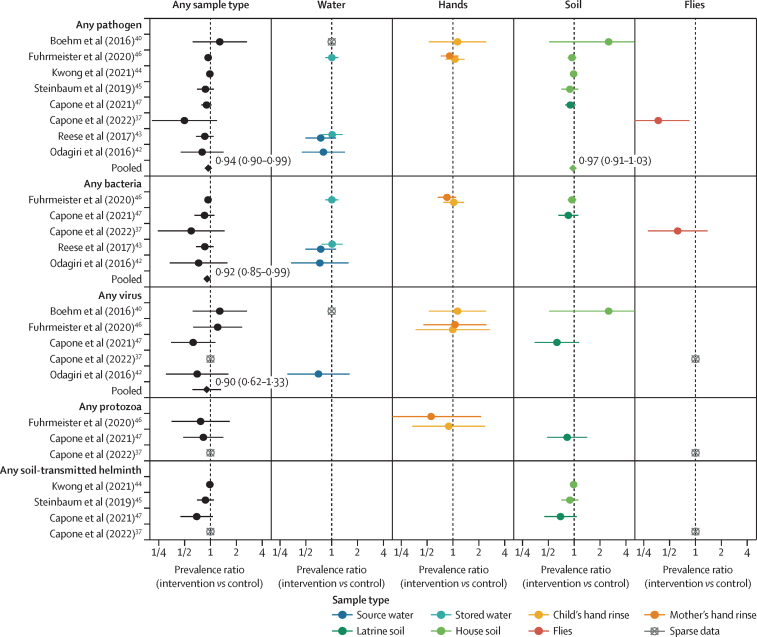
Forest plots of water, sanitation, and hygiene intervention effects on the prevalence of any enteropathogen or type of enteropathogen (any bacteria, virus, protozoa, or soil-transmitted helminth) in different types of environmental samples Pooled estimates are presented when there are four or more study-specific estimates for a specific sample type and target combination and are denoted with diamond-shaped points. Grey crossed points denote data that were too sparse to estimate a prevalence ratio (ie, less than ten positive observations). Samples of the same type from different locations (source *vs* stored water, flies in kitchen *vs* latrine, and soil from courtyard *vs* latrine) or different individuals (children's *vs* mothers' hands) are plotted separately. Point estimates and confidence intervals are given next to pooled estimates. Study-specific effects are independently estimated for each individual study, adjusting for potential confounders, and then pooled across studies with random effects models.

**Figure 3:**
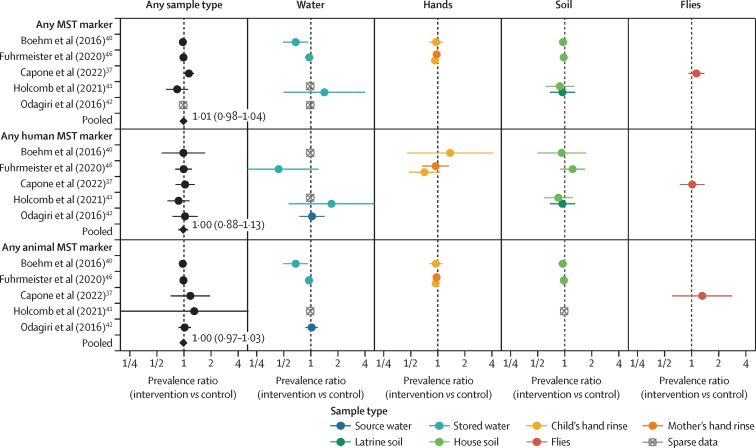
Forest plots of water, sanitation, and hygiene intervention effects on the prevalence of any MST marker or type of MST marker (human or animal MST markers) in different types of environmental samples Pooled estimates are presented when there are four or more study-specific estimates for a specific sample type and target combination and are denoted with diamond-shaped points. Grey crossed points denote data that were too sparse to estimate a prevalence ratio (ie, less than ten positive observations). Samples of the same type from different locations (source *vs* stored water, flies in kitchen *vs* latrine, and soil from courtyard *vs* latrine) or different individuals (children's *vs* mothers' hands) are plotted separately. Point estimates and confidence intervals are given next to pooled estimates. Study-specific effects are independently estimated for each individual study, adjusting for potential confounders, and then pooled across studies with random effects models. MST=microbial source tracking.

Interventions decreased the prevalence of any pathogen in any sample type in most individual studies but confidence intervals for PRs often crossed the null (figure 2). Among individual sample types, pathogen prevalence was most markedly reduced in flies in Capone and colleagues (adjusted PR 0·37 [95% CI 0·16–0·85]; figure 2).^37^ Pooled across studies, there was a small reduction in the prevalence of any pathogen detected in any sample type (pooled adjusted PR 0·94 [95% CI 0·90–0·99]; figure 2). Intervention effects on MST markers were inconsistent and mostly small or null across all sample types, with a null pooled effect (1·01 [0·98–1·04]; figure 3).

Interventions reduced the prevalence of any bacterial pathogens in any sample type (pooled adjusted PR 0·92 [95% CI 0·85–0·99]) and the intervention effects were protective in all individual studies, although with varying precision (figure 2). Interventions did not reduce virus prevalence in any sample type, with a pooled adjusted PR of 0·90 (0·62–1·33) and inconsistent directions of effects across individual studies, or reduce virus prevalence within specific sample types (figure 2). Intervention effects in individual studies were generally in the protective direction for protozoa and helminths but we did not have sufficient studies to pool estimates. Among specific pathogens, interventions had the strongest effects on the prevalence of adenovirus (0·21 [0·06–0·68]) and *Shigella* (0·28 [0·10–0·78]) in any sample type in Capone and colleagues,^47^ driven by reductions in soil around latrines (appendix p 2).

Overall, intervention effects on MST markers were negligible, with no detected effects on both human markers (pooled adjusted PR 1·00 [95% CI 0·88–1·13]) and animal markers (1·00 [0·97–1·03]; figure 3, appendix p 3). Interventions had the largest effects on any animal marker in stored water in Boehm and colleagues^40^ (adjusted PR 0·69 [95% CI 0·50–0·95]; figure 3), driven by a reduction in the ruminant BacR marker (0·62 [0·43–0·90]; appendix p 3), and on the human marker HF183 in any sample type (0·67 [0·48–0·95]; appendix p 3) in Holcomb and colleages.^41^

Of all observations, 20 125 (50%) of 40 224 had abundances quantified, including STH egg counts^44^, ^45^ and gene copies of enteropathogens and MST targets^37^, ^40^, ^46^ (appendix pp 4–5). Of these, 5377 (27%) of 20 187 were below the specific study-reported LOD, 5875 (29%) of 20 187 below the study-reported LOQ, and 8884 (44%) of 20 187 within the study-reported ROQ. Of targets enumerated within specific sample types, only 8 (18%) of 45) had more than 50% of samples within the ROQ and were included in our analysis. The abundance of the BacCow animal marker was lower in mothers' hand rinses in the sanitation intervention group in Fuhrmeister and colleagues,^46^ with an adjusted log_10_-transformed difference of –0·28 (95% CI –0·49 to –0·07) per pair of hands (table 2). The interventions had inconsistent effects on the abundance of other MST targets and STH egg counts, with confidence intervals crossing the null (table 2).

**Table 2:** Mean abundances of enteropathogen and MST targets by study group

	**Sample**	**Target**	**Sample**	**Range of quantification**	**Control**	**Intervention**	**Intervention effect (95% CI)**	**p value**	Wilcoxon p value*
Capone et al (2022)^37^	Latrine soil	Human (BacHum)	173	77·5%	3·8 (1·3), 3·8 (2·4–4·5)	4·0 (0·9), 4·2 (3·4–4·6)	0·14 (−0·19 to 0·47)	0·41	0·07
Holcomb et al (2021)^41^	Latrine soil	Human (*Methanobrevibacter smithii*)	113	51·3%	6·7 (0·6), 6·5 (6·2–7·0)	6·5 (0·5), 6·3 (6·3–6·6)	−0·14 (−0·38 to 0·11)	0·27	0·58
Kwong et al (2021)^44^	House soil	*Ascaris*	1426	100·0%	2·3 (6·7), 0·7 (0·0–2·5)	2·2 (6·9), 0·6 (0·0–2·3)	0·97 (0·68 to 1·38)†	0·85	0·54
Kwong et al (2021)^44^	House soil	*Trichuris*	1426	100·0%	1·6 (5·0), 0·4 (0·0–1·8)	2·0 (5·0), 0·4 (0·0–2·2)	1·22 (0·87 to 1·71)†	0·26	0·17
Steinbaum et al (2019)^45^	House soil	*Ascaris*	2101	100·0%	2·2 (18·8), 0	1·4 (9·3), 0	0·65 (0·33 to 1·28)†	0·21	0·33
Steinbaum et al (2019)^45^	House soil	*Trichuris*	2102	100·0%	0·2 (1·8), 0	0·2 (1·0), 0	0·73 (0·36 to 1·48)†	0·38	0·39
Fuhrmeister et al (2020)^46^	Child's hand rinse	Animal (BacCow)	365	75·9%	3·6 (1·4), 3·9 (3·5–4·4)	3·4 (1·4), 3·8 (1·2–4·2)	−0·17 (−0·47 to 0·12)	0·25	0·17
Fuhrmeister et al (2020)^46^	Mother's hand rinse	Animal (BacCow)	725	66·5%	3·3 (1·4), 3·8 (1·2–4·3)	3·0 (1·5), 3·7 (1·2–4·1)	−0·28 (−0·49 to −0·07)	0·01	0·01

Data are n, %, mean (SD), and median (IQR). Means are log_10_-transformed gene copies for MST markers and mean egg counts for soil transmitted helminths (*Ascaris* and *Trichuris*). Intervention effects are shown as adjusted differences in log_10_-transformed gene copies and ratios of helminth egg counts between the intervention and control arms. MST=microbial source tracking.

* Non-parametric Wilcoxon rank sum test p value.

† Marks ratio estimates from negative binomial model.

Intervention effects differed by season, but the direction of effects was inconsistent (appendix pp 5–6). Animal ownership was high (>80%) in all studies except Gram Vikas (appendix pp 39–42), but there were no consistent differences in intervention effects when households were stratified by animal presence (appendix p 7), and no differences in intervention effects on pathogens with possible zoonotic transmission versus only human hosts (appendix p 8). In Wald tests, there were no significant differences in pooled estimates between the one urban study (MapSan) and the four rural studies (p=0·25), between randomised and quasi-experimental studies (p=0·43), between studies with 1 year or less and more than 1 year of follow-up (p=0·51) or between the four studies with high latrine access among intervention recipients compared with the TSC trial with lower access (p=0·57). Adjustment covariates were measured differently across studies, but most had low missingness when measured (appendix pp 39–42). Unadjusted and adjusted estimates were similar (appendix pp 10–11), and results were also similar with bootstrapped LASSO penalised regression models to select adjustment covariates (appendix p 12).

## Discussion

Our IPD analysis of five intervention studies, mostly focused on household-level and community-level onsite sanitation improvements, indicates a small overall reduction in pathogen prevalence in the environment associated with the interventions. Although the effects of interventions on pathogen prevalence within individual studies had variable precision, point estimates of intervention effects were consistently in the protective direction across studies when aggregated across pathogen and sample types, despite differences in setting, intervention design, and length of follow-up. In general, intervention effects were less consistent when disaggregated by pathogen and sample type, with confidence intervals overlapping with the null (figure 2), but interventions were consistently protective against bacterial pathogens and for source water, and the MapSan study consistently showed protective effects. Interventions generally did not reduce the prevalence of human or animal faecal markers in included studies (figure 3).

These findings add to a body of literature on the effectiveness of sanitation improvements in low-income countries in interrupting faecal-oral transmission. A previous systematic review found no effect of sanitation interventions on FIB in the environment.^7^ The small, pooled effect on pathogens in the environment in our analysis indicates that any reductions in pathogen transmission through environmental pathways was most likely small. This can help explain the null findings of the parent trials on child diarrhoea.^3^, ^4^, ^6^, ^34^, ^35^ Among the five included studies, only WASH Benefits Bangladesh found a significant reduction in diarrhoea^3^ and a reduction in parasite infections^48^, ^49^ in the sanitation group compared with the controls. Diarrhoea was reduced by 2·2 percentage points on the absolute scale, compatible with a small reduction in pathogen transmission. Taken together, these findings indicate that the sanitation interventions in the studies in our review did not sufficiently isolate faecal waste from the environment, despite most of them achieving high levels of latrine access and use by adults. Young children's faeces are a dominant source of faecal contamination in the household environment,^50^ whereas animal faeces make up the majority of global faecal waste^51^ and are associated with increased domestic contamination.^52^ Therefore, containment of adult human waste might be insufficient to reduce environmental contamination in settings with continued child open defecation and high exposure to animal waste.^53^ Only the WASH Benefits trials included tools for child and animal faeces management (potties and scoops).^39^, ^54^ Notably, in two studies nested within WASH Benefits Bangladesh, we found reduced prevalence of ruminant (BacR) markers in stored water and reduced abundance of animal markers (BacCow) on mothers' hands (appendix p 3). Also, the sanitation intervention in this trial only reduced pathogen prevalence in households with animals (appendix p 7). The reduction in animal faecal contamination can help explain the unique health effects in this trial. In our analysis, only the MapSan study had a reduction in a human (HF183) marker. More comprehensive sanitation programmes, such as safely managed sanitation services that include safe removal in addition to containment of faecal waste, and interventions targeting child and animal faeces, can potentially more effectively interrupt environmental pathogen transmission.^55^

It is possible that current approaches to environmental sampling have affected the ability to detect intervention effects on pathogen presence in the environment. Faecal contamination in the domestic environment varies spatially and temporally,^56^, ^57^ and pathogen presence in the environment is intermittent, depending on the presence of infected individuals, shedding rates and pathogen fate and survival in environmental reservoirs.^58^ Different pathogens have different predominant transmission pathways, and specific pathogens might cause illness through a particular pathway too infrequently to capture with cross-sectional grab samples. Additionally, pathogen prevalence and abundance in the environment is typically low,^58^ and in some cases too rare to estimate the effects of interventions. Any reductions in pathogen presence might be more apparent with larger sample sizes or repeated sampling with high temporal and spatial resolution, which is costly for available pathogen detection methods, or by analysing larger quantities of composite samples. In addition, human MST markers have low specificity and sensitivity in settings with widespread faecal contamination in the environment.^28^, ^59^ Also, molecular methods for pathogen detection do not provide information on viability, and the clinical implications of small amounts of pathogen DNA or RNA detected in a sample are unclear. Although FIB have limitations in terms of low specificity to faecal sources and poor correlation with pathogens, culture-based FIB enumeration captures viable organisms, and large numbers of temporal or spatial samples can be analysed at low cost. Therefore, studies evaluating the environmental effect of WASH interventions can benefit from combining molecular pathogen measurements with culture-based FIB measurements to leverage the different strengths of these approaches. Pathogen-specific testing can supplement FIB data to identify the specific causes through which WASH interventions improve health or the effects of targeted interventions on specific pathogens. Advances in technology that reduce the costs of molecular diagnostics or increased funding for environmental testing within WASH trials might allow broader use of pathogen detection methods to estimate intervention effects on environmental contamination more precisely.

Our analysis had some strengths and limitations. The IPD meta-analysis allowed us to estimate intervention effects across studies with consistent statistical methodology, variable definitions, and covariate selection.^60^ The individual studies in our review were designed and powered to detect effects on child health, and samples were collected and analysed to detect pathogens and MST markers among smaller subsets of study households. Pooling effect estimates across studies increased our statistical power to detect a small overall effect on pathogens that individual studies were underpowered to detect. However, pooling assumes that individual studies are sufficiently homogeneous despite implementing different interventions in different settings. Therefore, pooled estimates should be interpreted in conjunction with estimates from individual studies. In our analysis, we detected no statistical heterogeneity between studies, and low-precision estimates from individual studies were qualitatively aligned with high-precision pooled estimates, suggesting that pooling data did not obscure any study-specific trends. Similarly, because studies measured different targets in different environmental matrices and many targets were detected infrequently, we relied on composite measures, such as detection of any pathogen in any sample type, to allow pooling effect estimates across studies. These measures combine data on different pathogens in different sample types and obscure nuances on which specific pathogens along which pathways are influenced by interventions. Therefore, effects on these composite outcomes should be interpreted in tandem with pathogen-specific and pathway-specific estimates. Studies assessing the effects of WASH interventions on environmental contamination should strive to have sample sizes that allow estimating intervention effects on pathogen prevalence and abundance in environmental matrices with precision, informed by a priori power calculations. Standardised measurement and reporting of a harmonised panel of enteropathogens in a consistent set of environmental matrices can also allow better comparability of pathogen-specific data for future IPD meta-analyses.^15^

Only a small number of studies met our inclusion criteria, limiting the generalisability of our findings. Three of the studies were from rural southeast Asia, with a defined monsoon season coinciding with peak diarrhoeal disease incidence, one from rural Kenya with two distinct rainy seasons, and one from drier and urban Maputo, Mozambique. This is consistent with previous WASH reviews indicating that Bangladesh, India, and Kenya are well represented in the published literature, and evidence from other settings is scarce.^61^ Four of the included studies focused on onsite sanitation and one evaluated a combined piped water and sanitation intervention. Therefore, we were unable to explore the effects of individual water supply and treatment and hygiene interventions, and more comprehensive sanitation modalities that safely managed sanitation services and sewer connections. The one study identified by the systematic review, but in which data were not shared, was a combined handwashing and water treatment intervention that reduced *V cholerae* in stored water but not source water.^37^ This is in contrast to the null effects of the Gram Vikas and TSC interventions on *V cholerae* in drinking water, and including this study in our IPD analysis would have contributed an additional effect estimate in which the intervention reduced pathogen prevalence. However, the study was done in high-risk populations (household contacts of cholera patients) and is not directly comparable to the studies included in our analysis that focused on general paediatric populations. Additionally, although the studies tested a diverse set of sample types, including understudied pathogen reservoirs, such as soil, not all pathways were captured. For example, contaminated food has been identified as a dominant pathogen transmission pathway in previous research,^62^ but only one study in our review sampled food and none of the three that tested MST markers were detected above our data sparsity cutoff to estimate treatment effects.

The basic sanitation interventions in our review resulted in a small reduction in the environmental presence of enteropathogens, consistent with the previously reported scarce health effects. Our results suggest that these sanitation interventions failed to contain human waste and thus prevent exposure to enteropathogens in these populations. More comprehensive approaches are needed to catalyse major health gains. Countries that have universal access to effective sanitation have seen remarkable improvements in health.^63^ Public health programmes in low-income countries should pursue transformative WASH approaches that encompass the full chain of excreta management, including safe removal rather than mere containment and address child and animal faeces to interrupt environmental pathogen transmission more effectively. Also, our review indicates a scarcity of water supply, water quality, and hygiene trials that measured pathogens in the environment. Future studies should assess the effect of such interventions on environmental contamination by measuring a harmonised set of pathogens with adequately powered sample sizes and by measuring FIB more frequently in time and space to capture variability, and include understudied pathways, such as soil, food, and flies.

## Data sharing

The de-identified, aggregated individual participant data and data dictionary can be shared by request of investigators and after approval by the original trial data contributors. Data inquiries can be directed to Andrew Mertens at amertens@berkeley.edu. The statistical analysis code is available at https://github.com/amertens/wash-ipd.

## Declaration of interests

JER reports that a portion of her salary is supported by an unrestricted donation to The London School of Hygiene & Tropical Medicine from Reckitt; the salary support is wholly unrelated to her role in the preparation of this manuscript. TC has interest in publicly traded companies. All other authors declare no competing interests.
