# Supplementary material for: Effects of water, sanitation, and hygiene interventions on detection of enteropathogens and host-specific faecal markers in the environment: a systematic review and individual participant data meta-analysis
Source: Lancet Planet Health. 2023 Mar 6;7(3):e197–208. doi: 10.1016/S2542-5196(23)00028-1 (PMC10009758; doi:10.1016/S2542-5196(23)00028-1)
Supplement: Supplementary appendix [file mmc1.pdf]

THE LANCET
Planetary Health

Supplementary appendix

This appendix formed part of the original submission and has been peer reviewed. We post it as supplied by the authors.

Supplement to: Mertens A, Arnold BF, Benjamin-Chung J, et al. Effects of water, sanitation, and hygiene interventions on detection of enteropathogens and host-specific faecal markers in the environment: a systematic review and individual participant data meta-analysis. *Lancet Planet Health* 2023; **7:** e197–208.

Effect of water, sanitation, and hygiene interventions on detection of enteropathogens and host-specific fecal markers in the environment: an individual-participant data meta-analysis

Andrew Mertens PhD, Benjamin F. Arnold PhD, Jade Benjamin-Chung PhD, Prof Alexandria B. Boehm PhD, Joe Brown PhD, Drew Capone PhD, Prof Thomas Clasen PhD, Erica Fuhrmeister PhD, Jessica A. Grembi PhD, David Holcomb PhD, Jackie Knee PhD, Laura H Kwong PhD, Audrie Lin PhD, Prof Stephen P. Luby MD, Rassul Nala MPH, Prof Kara Nelson PhD, Sammy M. Njenga PhD, Clair Null PhD, Amy J. Pickering PhD, Mahbubur Rahman MBBS, Heather E. Reese PhD, Lauren Steinbaum PhD, Prof Jill Stewart PhD, Ruwan Thilakaratne MPH, Oliver Cumming PhD, Prof John M. Colford Jr., Ayse Ercumen PhD

SUPPLEMENTARY FIGURES AND TABLES

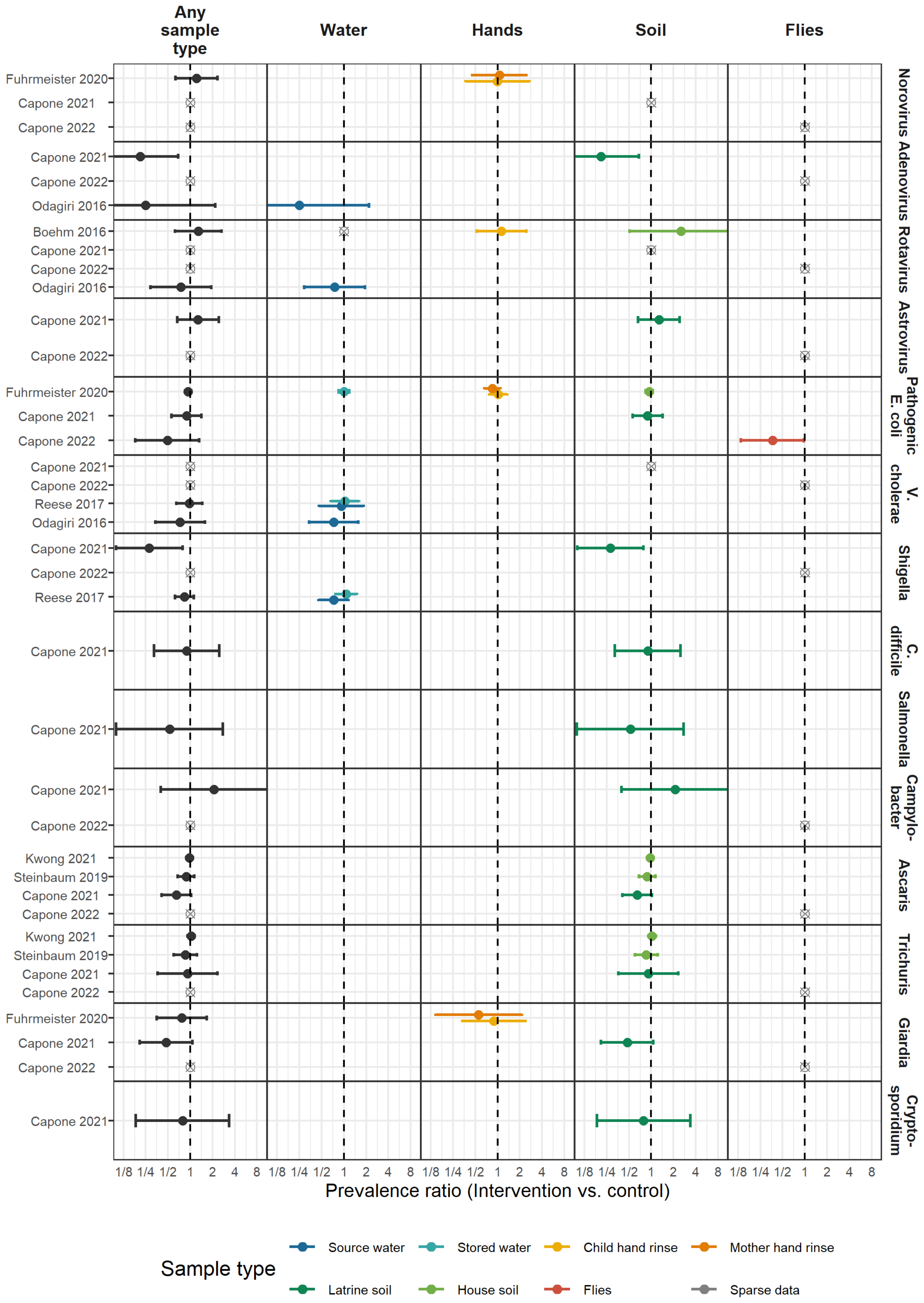

Figure S1. Forest plots of intervention effects on the prevalence of specific pathogens.

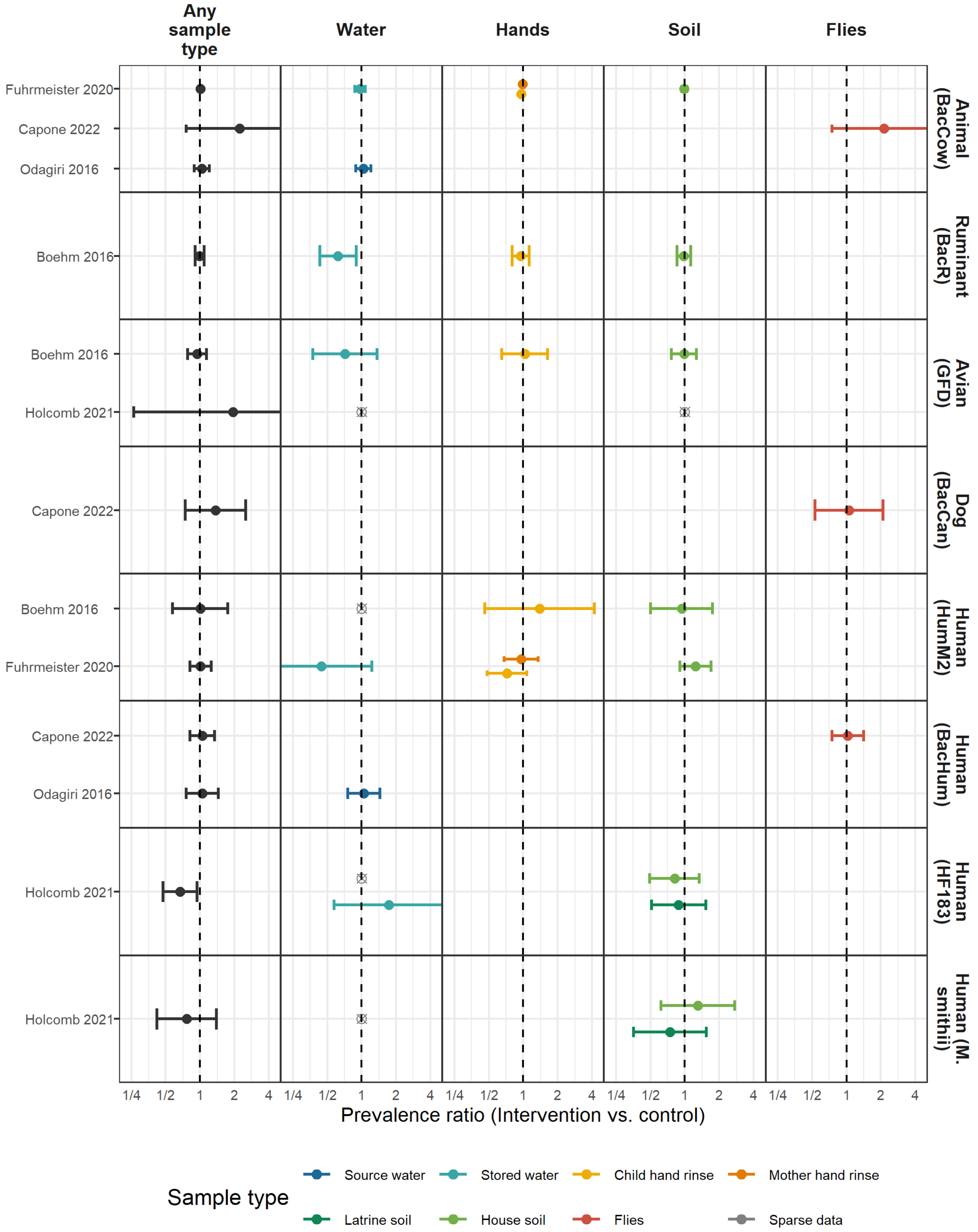

Figure S2. Forest plots of intervention effects on the prevalence of specific MST markers.

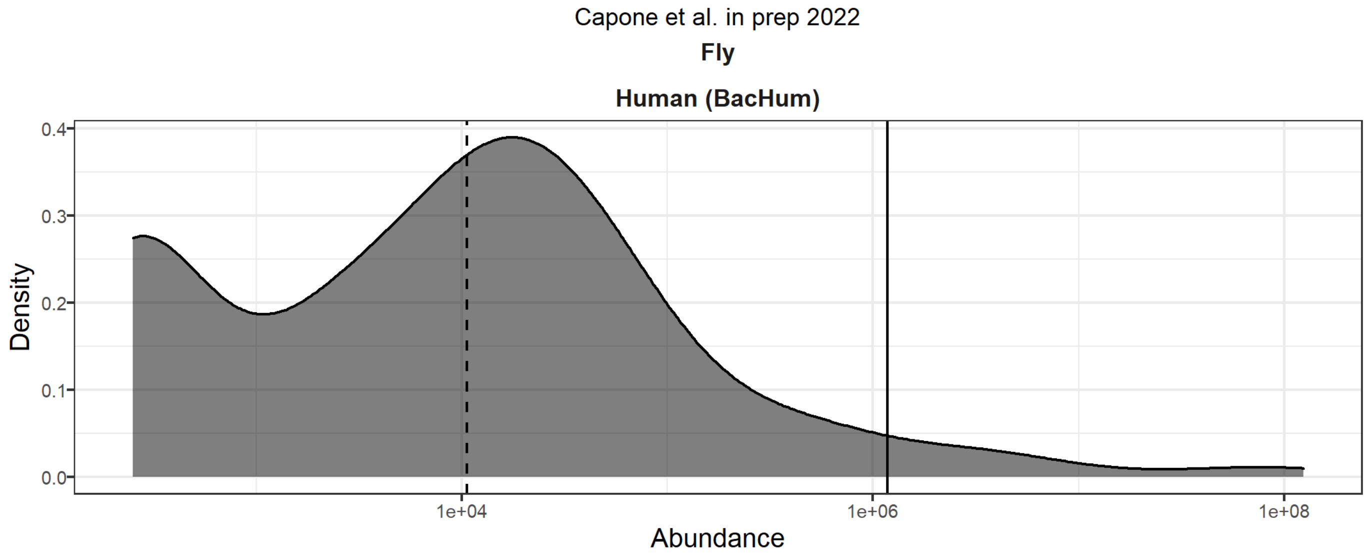
Capone et al. in prep 2022
Fly
Human (BacHum)
Density
0.0
0.1
0.2
0.3
0.4
1e+04
1e+06
1e+08
Abundance

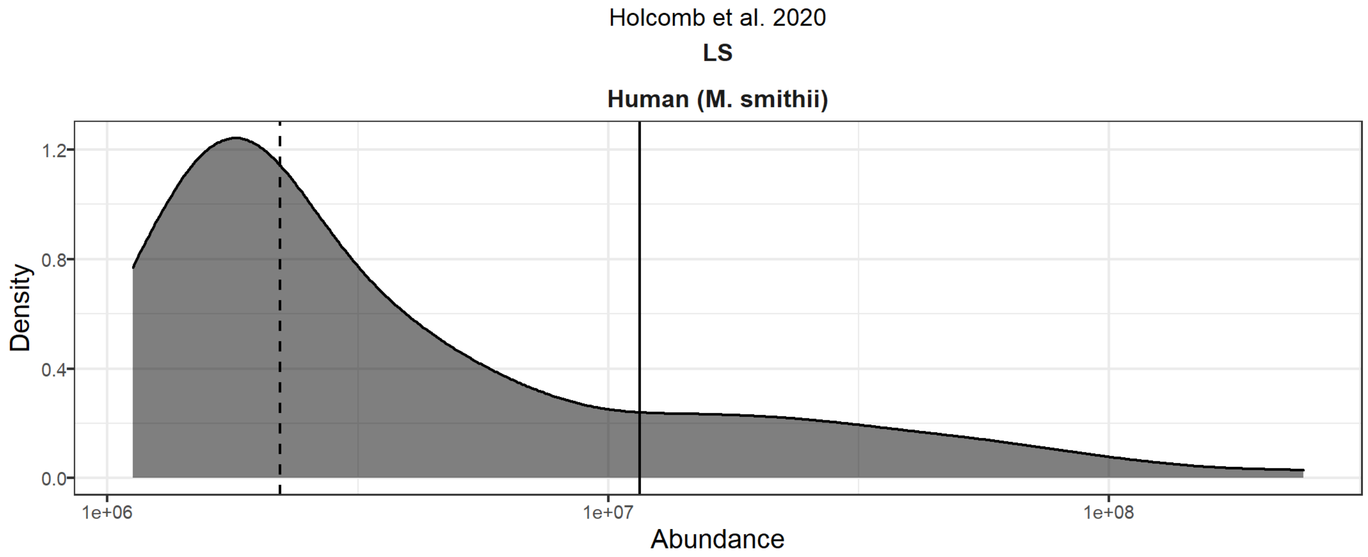
Holcomb et al. 2020
LS
Human (M. smithii)
Density
0.0
0.4
0.8
1.2
1e+06
1e+07
1e+08
Abundance

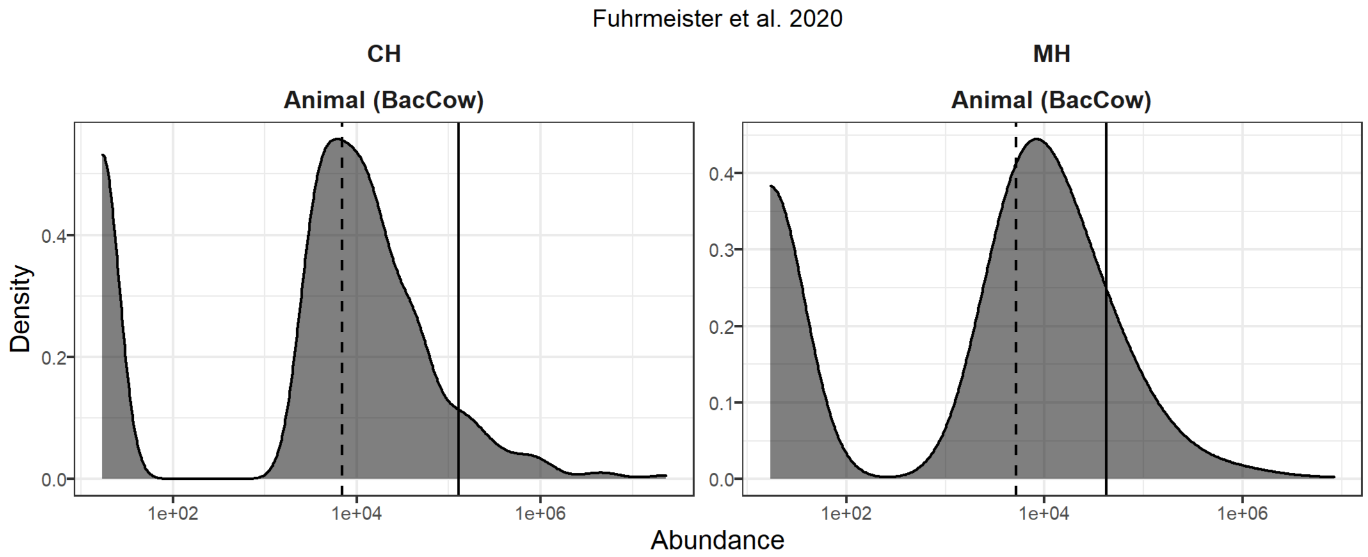
Fuhrmeister et al. 2020
CH
Animal (BacCow)
MH
Animal (BacCow)
Density
0.0
0.2
0.4
0.0
0.1
0.2
0.3
0.4
1e+02
1e+04
1e+06
1e+02
1e+04
1e+06
Abundance

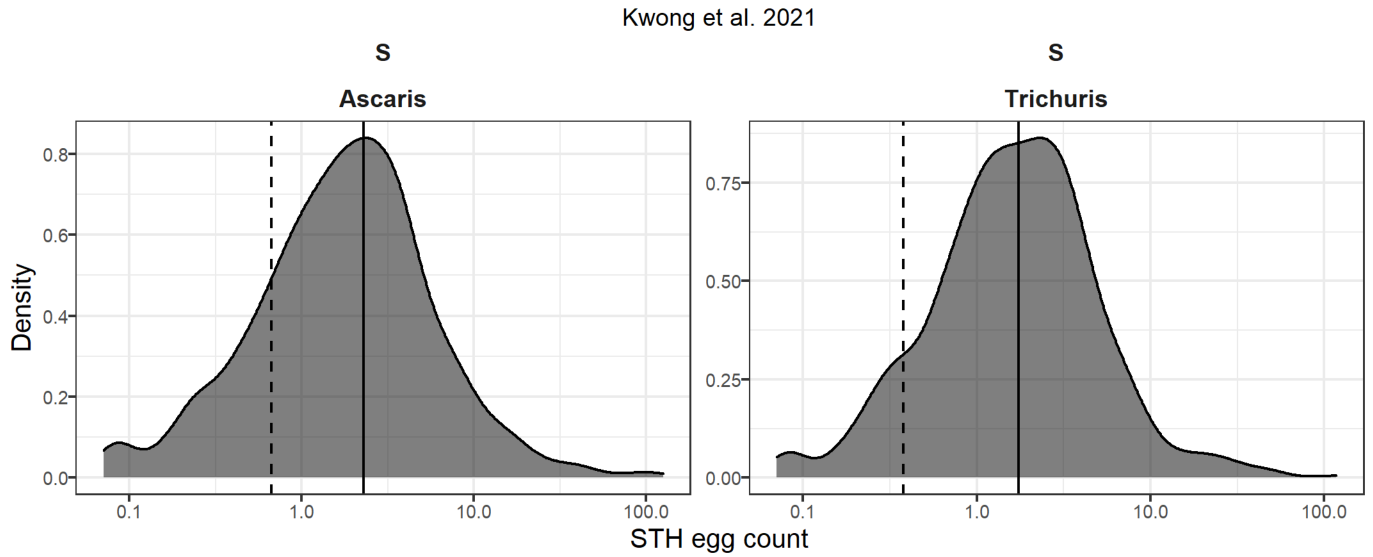

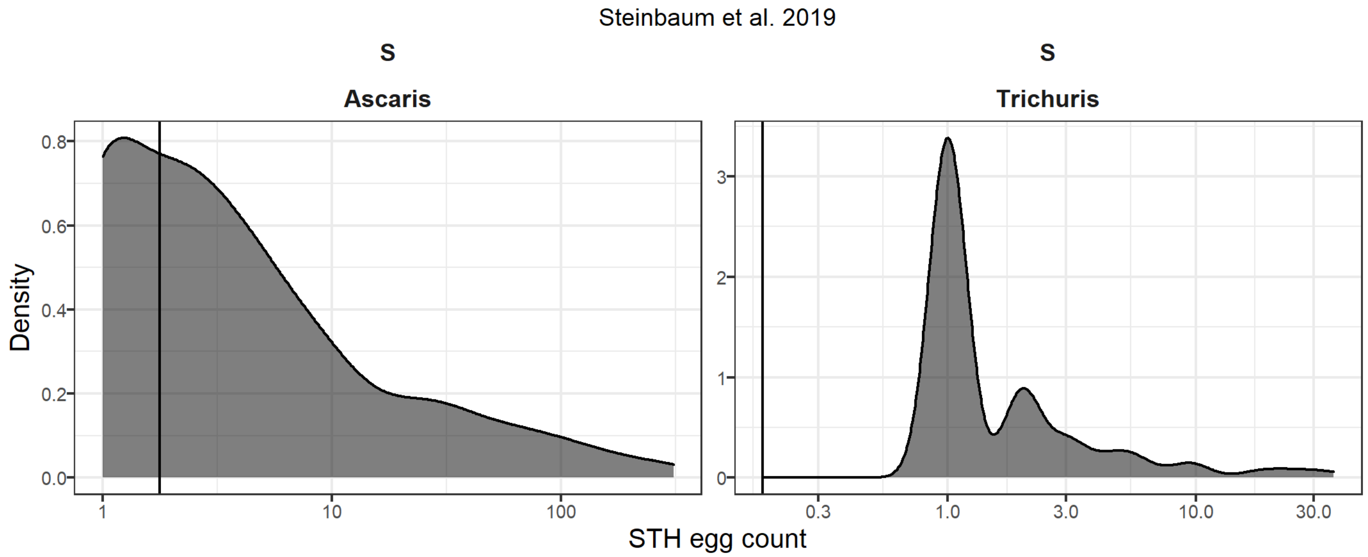

Figure S3. Distributions of abundance outcomes. The X-axes are displayed on the log-10 scale. Black vertical lines mark the means, and dashed lines mark the medians. Values below the limit of detection were imputed with with half the limit of detection and values below the limit of quantification were imputed with the midpoint between the limits of detections and quantification, leading to some bimodal distributions.

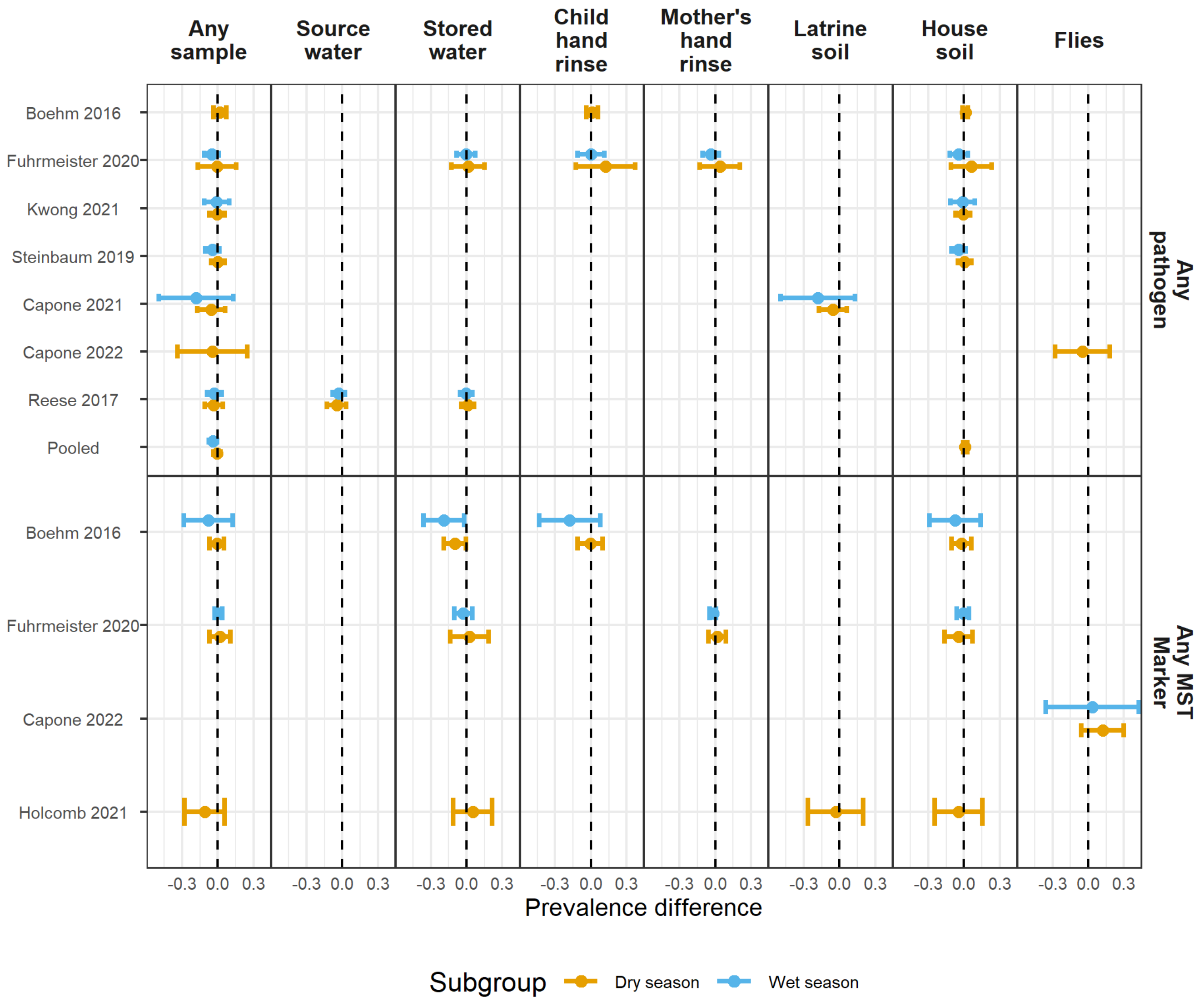

Figure S4. Forest plots of any enteropathogen prevalence differences or any MST prevalence differences between intervention and control arms, stratified by whether the sample was collected during the wet versus dry season (defined by the 6 months of highest average rainfall). Significant effect modification, as determined by the p-values on the regression model interaction term, is marked above points with asterisks (P < 0.05 = "*", P < 0.01 = "**", P < 0.001 = "***"). Grey crossed points denote data that were too sparse to estimate a prevalence ratio (i.e., <10 positive observations).

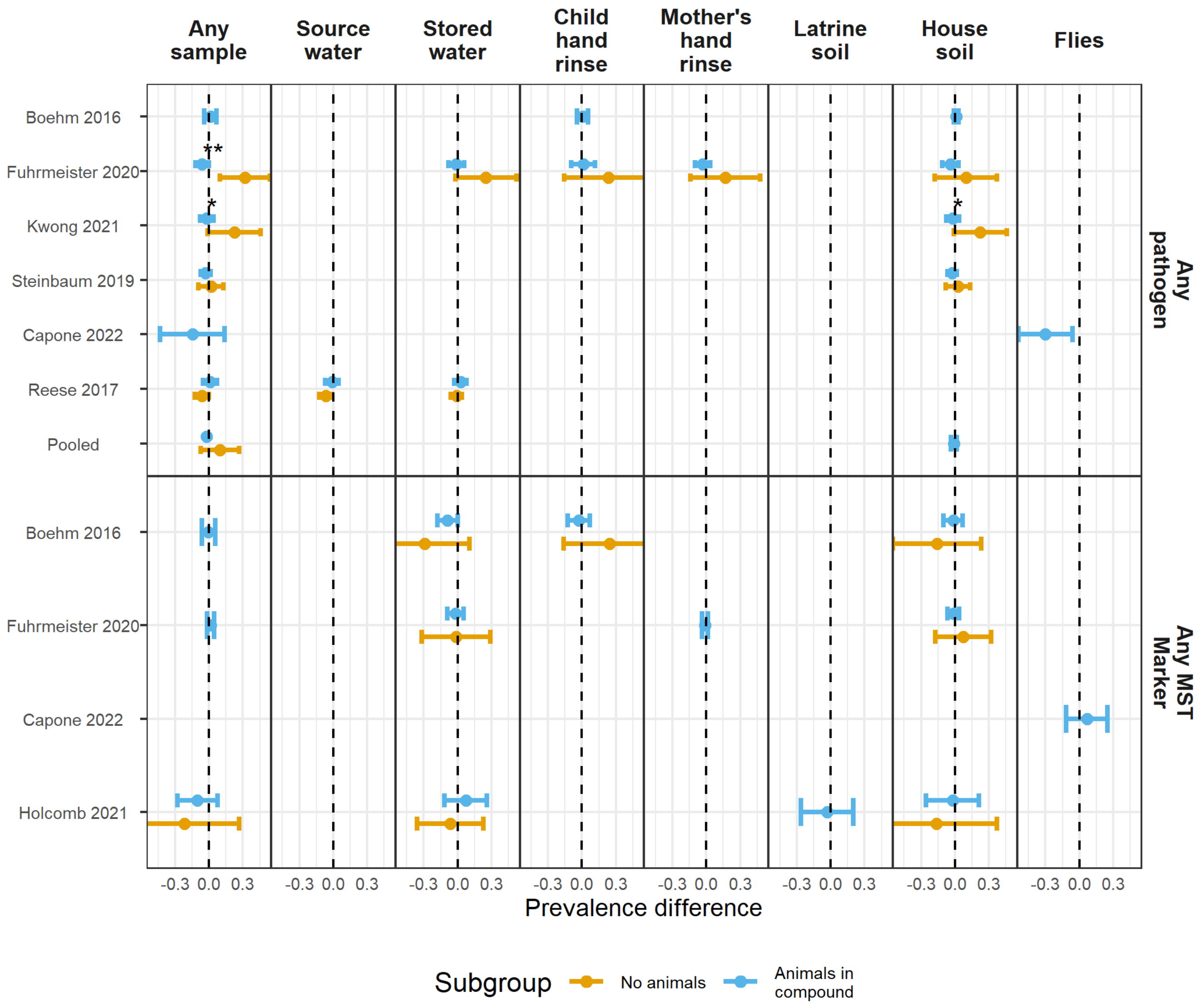

Figure S5. Forest plots of any enteropathogen prevalence differences or any MST prevalence differences between intervention and control arms, stratified by whether any animals were present in the compound. Significant effect modification, as determined by the p-values on the regression model interaction term, is marked above points with asterisks (P < 0.05 = "*", P < 0.01 = "**", P < 0.001 = "***"). Grey crossed points denote data that were too sparse to estimate a prevalence ratio (i.e., <10 positive observations).

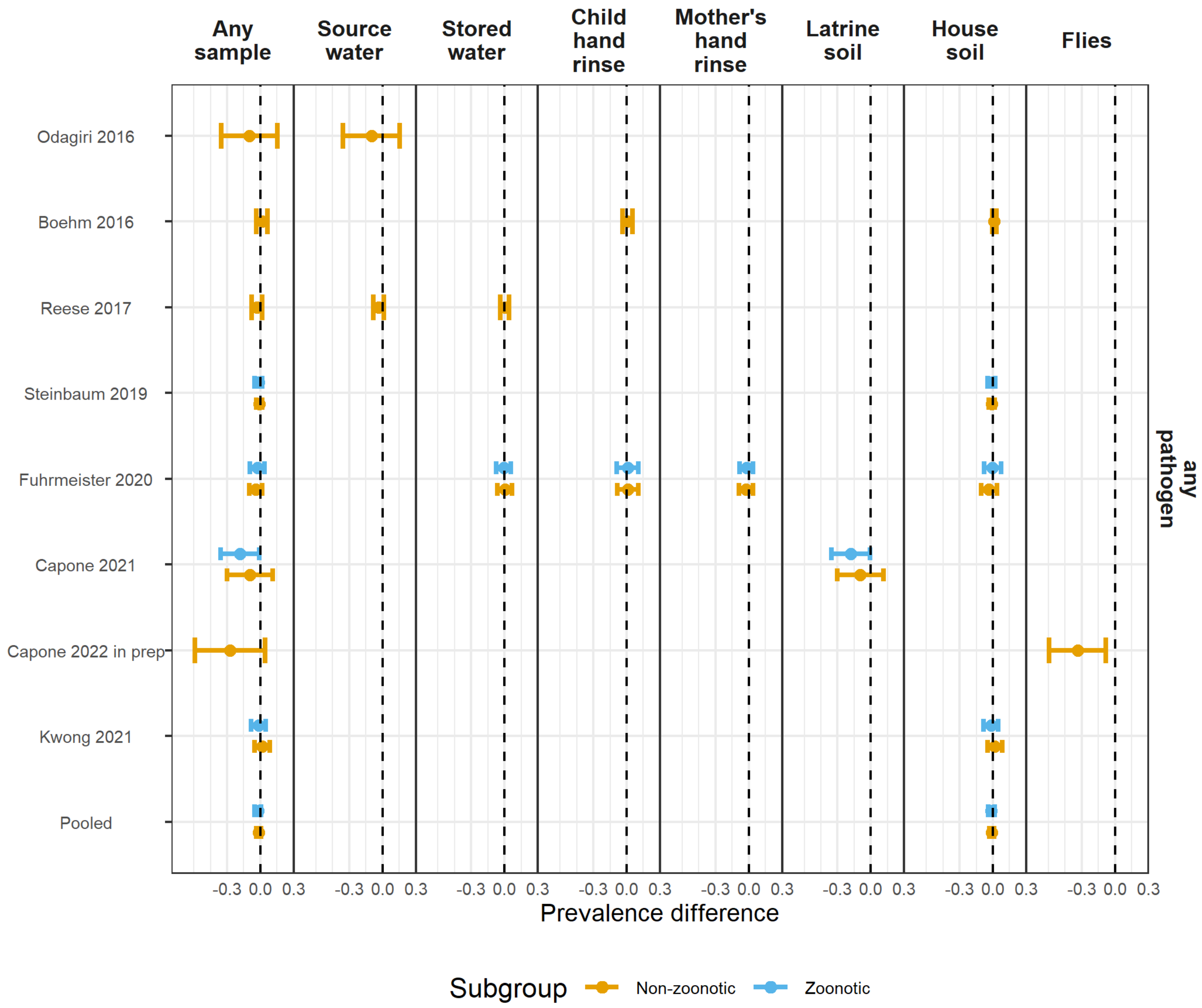

Figure S6. Forest plots of any enteropathogen prevalence differences or any MST prevalence differences between intervention and control arms, stratified by whether the pathogen is zoonotically transmitted. Grey crossed points denote data that were too sparse to estimate a prevalence ratio (i.e., <10 positive observations). Significant effect modification, as determined by the p-values on the regression model interaction term, is marked above points with asterisks (P < 0.05 = "*", P < 0.01 = "**", P < 0.001 = "***"). Grey crossed points denote data that were too sparse to estimate a prevalence ratio (i.e., <10 positive observations).

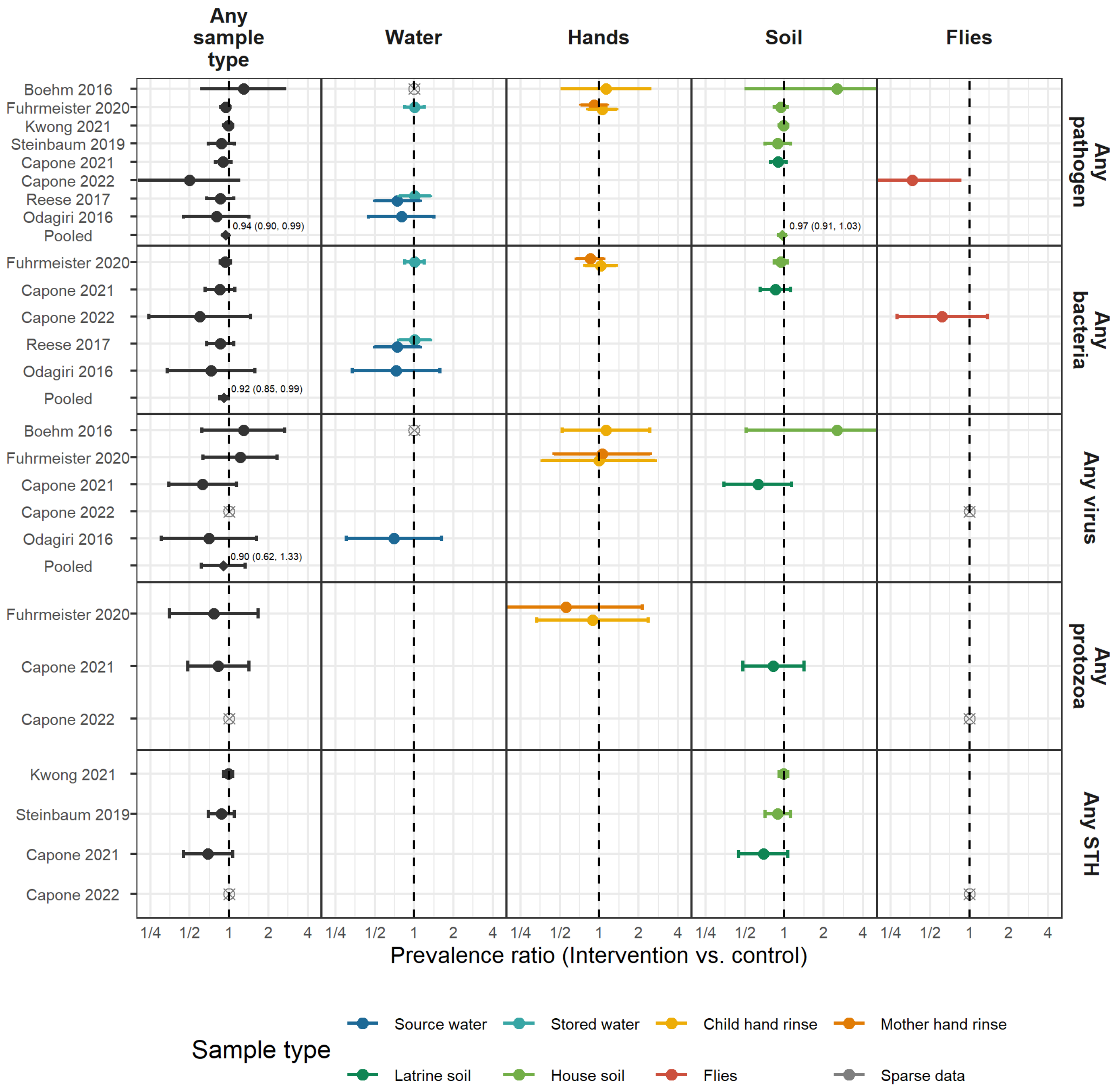

Figure S7. Forest plots of unadjusted intervention effects on the prevalence of any enteropathogen or type of enteropathogen (any bacteria, any virus, any protozoa and any STH) in different types of environmental samples. Point estimates and confidence intervals are printed next to pooled estimates. Grey crossed points denote data that were too sparse to estimate a prevalence ratio (i.e., <10 positive observations).

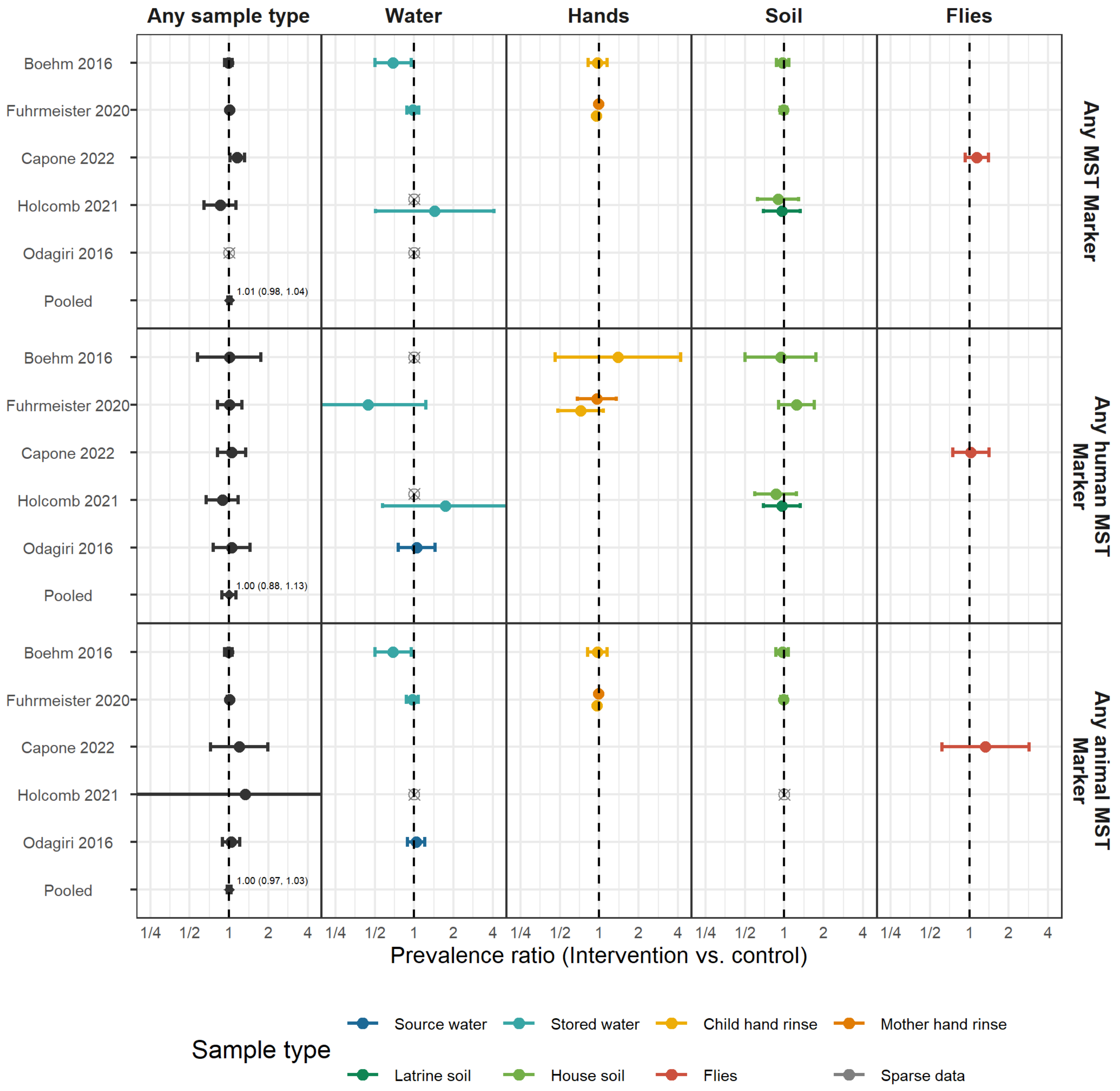

Figure S8. Forest plots of unadjusted intervention effects on the prevalence of any MST marker or type of MST marker (human or animal MST markers) in different types of environmental samples. Point estimates and confidence intervals are printed next to pooled estimates. Grey crossed points denote data that were too sparse to estimate a prevalence ratio (i.e., <10 positive observations).

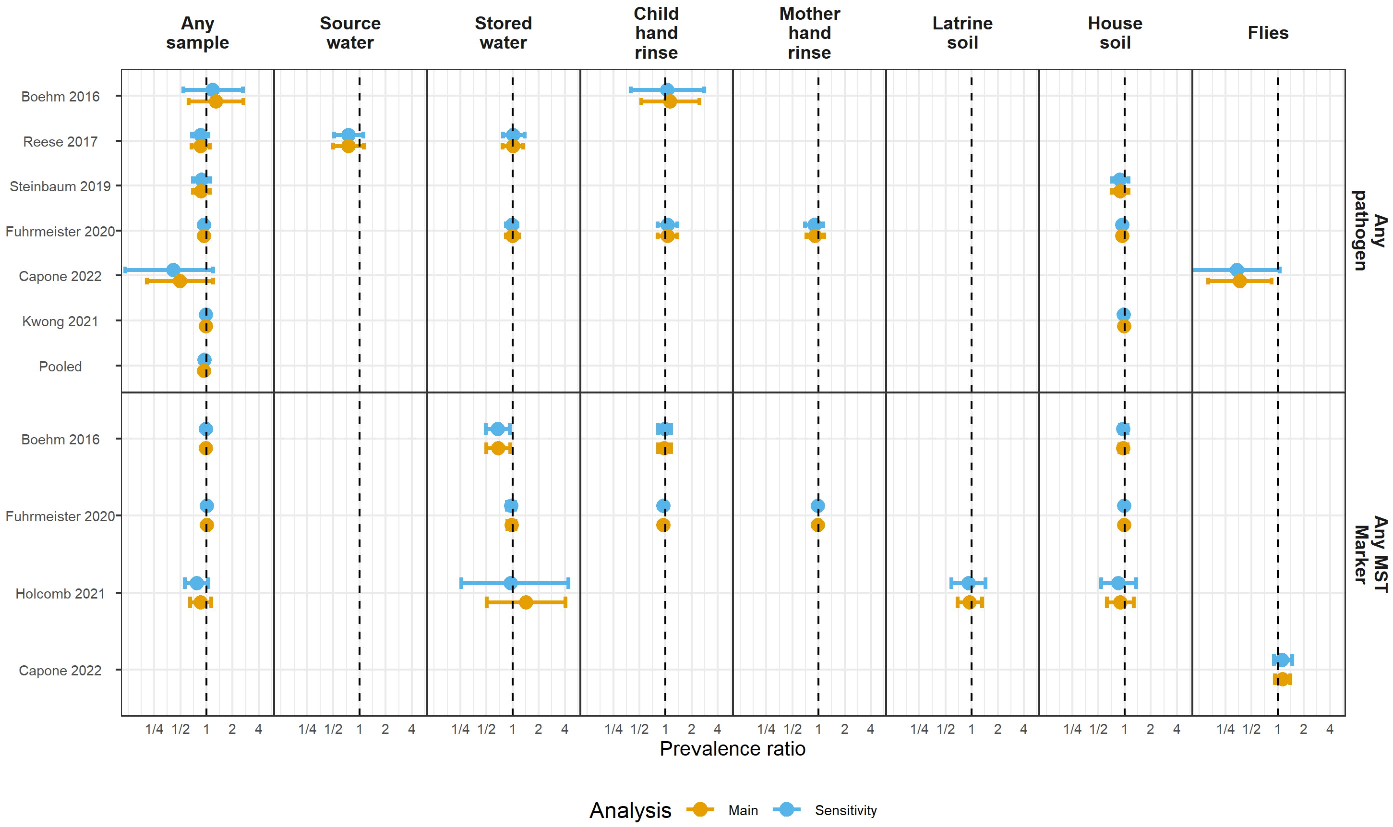

Figure S9. Forest plots of results from the primary analysis (orange) compared to a sensitivity analysis to the covariate prescreening method (blue) for the outcomes of any pathogen detection (top row) and any MST marker detection (bottom row) for different studies and sample types (columns). The sensitivity analysis used LASSO penalized regressions to select covariates across 200 bootstrap iterations with replacement, with 95% confidence intervals estimated with using the quantile method.

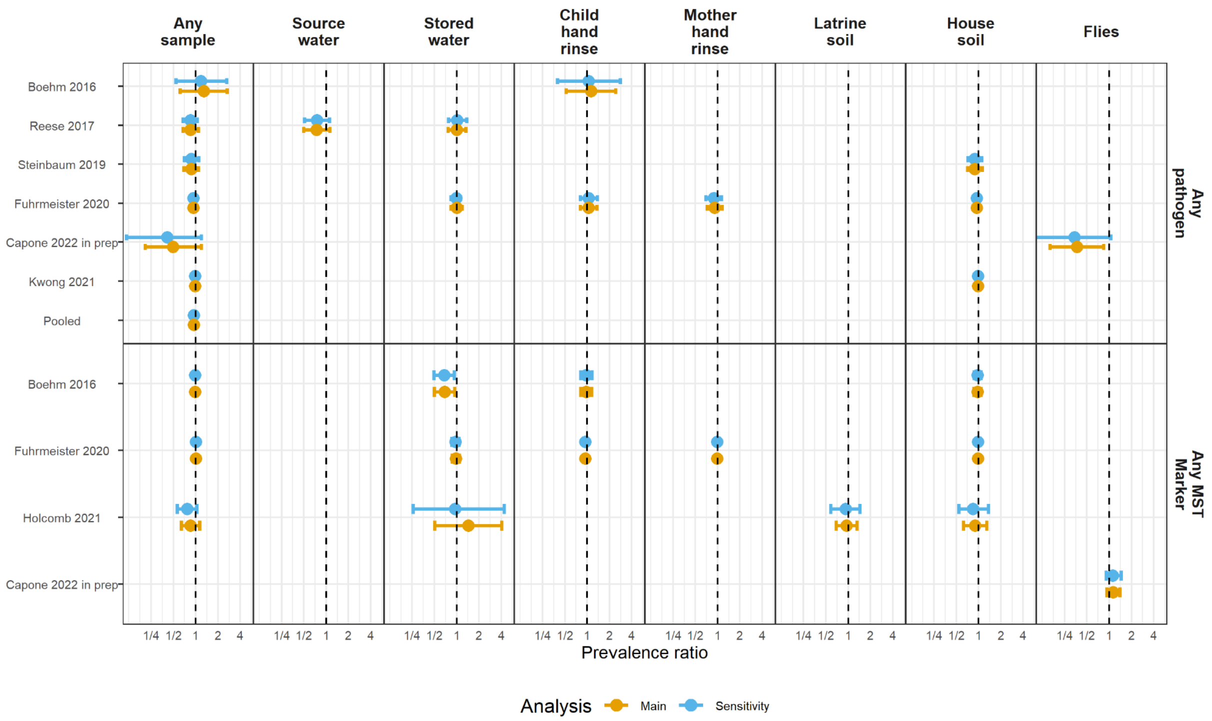

Figure S10. Forest plots of results from the primary analysis (orange) compared to a sensitivity analysis to the covariate prescreening method (blue) for the outcomes of any pathogen detection (top row) and any MST marker detection (bottom row) for different studies and sample types (columns). The sensitivity analysis used LASSO penalized regressions to select covariates across 200 bootstrap iterations with replacement, with 95% confidence intervals estimated with using the quantile method.

Table S1. Systematic review search terms

Search terms were combined with “OR” within columns and with “AND” across columns. We developed a search strategy from a two-step process. First, known key studies prior to the systematic review (WASH Benefits, Mapsan, Gram Vikas, Odisah [Table 1]) were examined for keywords and Medical Subject Heading (MeSH) terms relating to each of the following categories of terms comprising our search string: WASH interventions; microbial source tracking and environmental contamination; enteric infection; diarrhea; and child growth and development. Next, we performed an initial search using these terms and extracted other relevant terms and synonyms from relevant articles in the search results, resulting in the final list presented in this table.

Study design	WASH	Environmental markers	Child health
matched, trial, RCT, experiment, intervention, randomized, randomised, quasi-randomized, quasi-randomised, quasi-experimental, pseudo-randomized, pseudo-randomised, non-randomized controlled trials	Water, Sanitation, Hygiene, Handwashing, WSH, Sanitation, Water Supply, Sanitary Drainage, Toilet Facilities, Drinking Water, Hand Hygiene, Water Purification, Waste Water, disinfection	molecular source tracking, microbial source tracking, microbial transmission, diarrheal pathogen, diarrheal pathogens, diarrhoeal pathogen, diarrhoeal pathogens, fecal-oral, faecal-oral, entericpathogen, entericpathogens, ruminant, avian, Feces, Faeces, Fecal, Faecal, Fecally, Faecally	Entericinfection, Soil-transmitted helminth, Protozoan, Seroconversion, Fecal microbiology, Faecal microbiology, Fecal biomarker, Faecal biomarker, Intestinal Diseases, Parasitic, Seroconversion, Enteritis, Helminthiasis, Helminthiases, Intestinal infection, Viral infection, Bacterial infection, Parasite infection, Parasitic infection, Helminth infection, Fecal sampling, Faecal sampling, Stool sampling, Stool collection, Diarrhea, Dysentery, Child growth faltering, Growth faltering, Child development, Length-for-age, Height-for-age, Weight-for-age, Head circumference, Waist circumference, Stunting, Stunted, Wasting, Wasted, Linear growth, Anthropometric measurement, Malnutrition, Undernourished, Undernutrition, Underweight, Growth Disorders, Childnutrition disorder, Wasting syndrome, Thinness, Growth velocity

Table S2. Pubmed search string

[MH] are mesh headers and [TW] are text words. Search strings for other databases are available in the Open Science Framework analysis preregistration materials (https://osf.io/8sgzn/).

((matched [tw]) OR (trial [tw]) OR (RCT [tw]) OR (experiment [tw]) OR (intervention [tw]) OR (randomized [tw]) OR (randomised [tw]) OR (quasi-randomized [tw]) OR (quasi-randomised [tw]) OR (quasi-experimental [tw]) OR (pseudo-randomized [tw]) OR (pseudo-randomised [tw]) OR (“non-randomized controlled trials as topic” [mh])) AND ((Water [tw]) OR (Sanitation [tw]) OR (Hygiene [tw]) OR (Handwashing [tw]) OR (WSH [tw]) OR (“Sanitation” [mh]) OR (“Water Supply” [mh]) OR (“Drainage, Sanitary” [mh]) OR (Sanitary Drainage [tw]) OR (“Toilet Facilities” [mh]) OR (“Drinking Water” [mh]) OR (“Hand Hygiene” [mh]) OR (“Water Purification” [mh]) OR (“Waste Water” [mh]) OR (disinfect* [tw])) AND ((molecular source tracking [tw]) OR (microbial source tracking [tw]) OR (microbial transmission [tw]) OR (diarrheal pathogen [tw]) OR (diarrheal pathogens [tw]) OR (diarrhoeal pathogen [tw]) OR (diarrhoeal pathogens [tw]) OR (fecal-oral [tw]) OR (faecal-oral [tw]) OR (enteric pathogen [tw]) OR (enteric pathogens [tw]) OR (ruminant* [tw]) OR (avian* [tw]) OR (“Feces” [mh]) OR (Feces [tw]) OR (Faeces [tw]) OR (Fecal [tw]) OR (Faecal [tw]) OR (Fecally [tw]) OR (Faecally [tw])) AND (((Enteric infection* [tw]) OR (Soil-transmitted helminth* [tw]) OR (Protozoan* [tw]) OR (Seroconversion [tw]) OR (Fecal microbio* [tw]) OR (Faecal microbio* [tw]) OR (Fecal biomarker* [tw]) OR (Faecal biomarker* [tw]) OR (“Intestinal Diseases, Parasitic/epidemiology” [mh]) OR (“Seroconversion” [mh]) OR (Seroconversion [tw]) OR (“Enteritis/epidemiology” [mh]) OR (“Helminthiasis/complications” [mh]) OR (Helminthiasis [tw]) OR (Helminthiases)OR (“Helminthiasis/epidemiology” [mh]) OR (“Helminthiasis/prevention and control” [mh]) OR (Intestinal infection* [tw]) OR (Viral infection* [tw]) OR (Bacterial infection* [tw]) OR (Parasite infection* [tw]) OR (Parasitic infection* [tw]) OR (Helminth infection* [tw]) OR (Fecal sampling [tw]) OR (Faecal sampling [tw]) OR (Bacterial

infection* [tw]) OR (Parasite infection* [tw]) OR (Parasitic infection* [tw]) OR (Helminth infection* [tw]) OR (Fecal sampling [tw]) OR (Faecal sampling [tw]) OR (Stool sampling [tw]) OR (Stool collection [tw])) OR ((Diarrh* [tw]) OR (Dysentery [tw]) OR ("Diarrhea/epidemiology" [mh]) OR ("Diarrhea/etiology" [mh]) OR ("Diarrhea/prevention and control" [mh]) OR ("Diarrhea, Infantile" [mh]) OR ("Dysentery" [mh])) OR (Child growth faltering [tw]) OR (Growth faltering [tw])OR (Child development [tw]) OR (Length-for-age [tw]) OR (Height-for-age [tw]) OR (Weight-for-age [tw]) OR (Head circumference [tw]) OR (Waist circumference [tw]) OR (Stunt* [tw]) OR (Wasting [tw]) OR (Wasted [tw]) OR (Linear growth [tw]) OR (Anthropometric measurement* [tw]) OR (Maln* [tw]) OR (Undernourish* [tw]) OR (Undernutrition [tw]) OR (Underweight [tw]) OR ("Growth Disorders" [mh]) OR (Growth Disorders [tw]) OR ("Child nutrition disorders" [mh]) OR (Child nutrition disorder* [tw]) OR ("Malnutrition" [mh]) OR ("Wasting Syndrome" [mh]) OR (Wasting syndrome [tw]) OR ("Thinness" [mh]) OR (Thinness [tw]) OR (Growth velocity [tw]))

Table S3. PRISMA Checklist

Topic	No.	Item	Location where item is reported
**TITLE**			
**Title**	1	Identify the report as a systematic review.	Title
**ABSTRACT**			
**Abstract**	2	See the PRISMA for Abstracts checklist below	
**INTRODUCTION**			
**Rationale**	3	Describe the rationale for the review in the context of existing knowledge.	Introduction paragraph 2
**Objectives**	4	Provide an explicit statement of the objective(s) or question(s) the review addresses.	Introduction paragraph 3
**METHODS**			
**Eligibility criteria**	5	Specify the inclusion and exclusion criteria for the review and how studies were grouped for the syntheses.	Methods, paragraph 1
**Information sources**	6	Specify all databases, registers, websites, organisations, reference lists and other sources searched or consulted to identify studies. Specify the date when each source was last searched or consulted.	Methods, paragraph 1, Fig. S1
**Search strategy**	7	Present the full search strategies for all databases, registers and websites, including any filters and limits used.	Tables S1-S2
**Selection process**	8	Specify the methods used to decide whether a study met the inclusion criteria of the review, including how many reviewers screened each record and each report retrieved, whether they worked independently, and if applicable, details of automation tools used in the process.	Methods, paragraph 1

Topic	No.	Item	Location where item is reported
**Data collection process**	9	Specify the methods used to collect data from reports, including how many reviewers collected data from each report, whether they worked independently, any processes for obtaining or confirming data from study investigators, and if applicable, details of automation tools used in the process.	Methods, paragraph 1
**Data items**	10a	List and define all outcomes for which data were sought. Specify whether all results that were compatible with each outcome domain in each study were sought (e.g. for all measures, time points, analyses), and if not, the methods used to decide which results to collect.	Methods, paragraphs 2-3
	10b	List and define all other variables for which data were sought (e.g. participant and intervention characteristics, funding sources). Describe any assumptions made about any missing or unclear information.	Methods, paragraph 2-3
**Study risk of bias assessment**	11	Specify the methods used to assess risk of bias in the included studies, including details of the tool(s) used, how many reviewers assessed each study and whether they worked independently, and if applicable, details of automation tools used in the process.	Methods, paragraph 7
**Effect measures**	12	Specify for each outcome the effect measure(s) (e.g. risk ratio, mean difference) used in the synthesis or presentation of results.	Methods, paragraph 4
**Synthesis methods**	13a	Describe the processes used to decide which studies were eligible for each synthesis (e.g. tabulating the study intervention characteristics and comparing against the planned groups for each synthesis (item 5)).	Methods, paragraph 1
	13b	Describe any methods required to prepare the data for presentation or synthesis, such as handling of missing summary statistics, or data conversions.	Methods, paragraph 4-7
	13c	Describe any methods used to tabulate or visually display results of individual studies and syntheses.	Figure captions
	13d	Describe any methods used to synthesize results and provide a rationale for the choice(s). If meta-analysis was performed, describe the model(s), method(s) to identify the presence and extent of statistical heterogeneity, and software package(s) used.	Methods, paragraph 5

Topic	No.	Item	Location where item is reported
	13e	Describe any methods used to explore possible causes of heterogeneity among study results (e.g. subgroup analysis, meta-regression).	Methods, paragraph 6
	13f	Describe any sensitivity analyses conducted to assess robustness of the synthesized results.	Methods, paragraph 7
**Reporting bias assessment**	14	Describe any methods used to assess risk of bias due to missing results in a synthesis (arising from reporting biases).	Not applicable
**Certainty assessment**	15	Describe any methods used to assess certainty (or confidence) in the body of evidence for an outcome.	Not applicable
**RESULTS**			
**Study selection**	16a	Describe the results of the search and selection process, from the number of records identified in the search to the number of studies included in the review, ideally using a flow diagram.	Figure S1
	16b	Cite studies that might appear to meet the inclusion criteria, but which were excluded, and explain why they were excluded.	Results, paragraphs 1-2
**Study characteristics**	17	Cite each included study and present its characteristics.	Results, paragraphs 1-2, Table 1
**Risk of bias in studies**	18	Present assessments of risk of bias for each included study.	Table S4
**Results of individual studies**	19	For all outcomes, present, for each study: (a) summary statistics for each group (where appropriate) and (b) an effect estimate and its precision (e.g. confidence/credible interval), ideally using structured tables or plots.	Figures 1,2 S2-S3, S5-S8, Tables 2, S6-S9
**Results of syntheses**	20a	For each synthesis, briefly summarise the characteristics and risk of bias among contributing studies.	Not applicable
	20b	Present results of all statistical syntheses conducted. If meta-analysis was done, present for each the summary estimate and its precision (e.g. confidence/credible interval) and measures of statistical heterogeneity. If comparing groups, describe the direction of the effect.	Figures 1 ,2 S2-S3, S5-S8, Tables 2, S6-S9

Topic	No.	Item	Location where item is reported
	20c	Present results of all investigations of possible causes of heterogeneity among study results.	Results, paragraph 12
	20d	Present results of all sensitivity analyses conducted to assess the robustness of the synthesized results.	Results, paragraph 12
**Reporting biases**	21	Present assessments of risk of bias due to missing results (arising from reporting biases) for each synthesis assessed.	Not applicable
**Certainty of evidence**	22	Present assessments of certainty (or confidence) in the body of evidence for each outcome assessed.	Figures1 ,2 S2-S3, S5-S8, Tables 2, S6-S9
**DISCUSSION**			
**Discussion**	23a	Provide a general interpretation of the results in the context of other evidence.	Discussion, paragraphs 1-2
	23b	Discuss any limitations of the evidence included in the review.	Discussion, paragraphs 3-4
	23c	Discuss any limitations of the review processes used.	Discussion, paragraphs 3-4
	23d	Discuss implications of the results for practice, policy, and future research.	Discussion, paragraph 5
**OTHER INFORMATION**			
**Registration and protocol**	24a	Provide registration information for the review, including register name and registration number, or state that the review was not registered.	https://osf.io/8sgzn/
	24b	Indicate where the review protocol can be accessed, or state that a protocol was not prepared.	https://osf.io/8sgzn/
	24c	Describe and explain any amendments to information provided at registration or in the protocol.	Not applicable
**Support**	25	Describe sources of financial or non-financial support for the review, and the role of the funders or sponsors in the review.	Accompanying manuscript

Topic	No.	Item	Location where item is reported
**Competing interests**	26	Declare any competing interests of review authors.	Accompanying manuscript
**Availability of data, code and other materials**	27	Report which of the following are publicly available and where they can be found: template data collection forms; data extracted from included studies; data used for all analyses; analytic code; any other materials used in the review.	Accompanying manuscript

3

Topic	No.	Item	Reported?
**TITLE**			
**Title**	1	Identify the report as a systematic review.	Yes
**BACKGROUND**			
**Objectives**	2	Provide an explicit statement of the main objective(s) or question(s) the review addresses.	Yes
**METHODS**			
**Eligibility criteria**	3	Specify the inclusion and exclusion criteria for the review.	Yes
**Information sources**	4	Specify the information sources (e.g. databases, registers) used to identify studies and the date when each was last searched.	No
**Risk of bias**	5	Specify the methods used to assess risk of bias in the included studies.	Yes
**Synthesis of results**	6	Specify the methods used to present and synthesize results.	Yes
**RESULTS**			
**Included studies**	7	Give the total number of included studies and participants and summarise relevant characteristics of studies.	Yes

Topic	No.	Item	Reported?
**Synthesis of results**	8	Present results for main outcomes, preferably indicating the number of included studies and participants for each. If meta-analysis was done, report the summary estimate and confidence/credible interval. If comparing groups, indicate the direction of the effect (i.e. which group is favoured).	Yes
**DISCUSSION**			
**Limitations of evidence**	9	Provide a brief summary of the limitations of the evidence included in the review (e.g. study risk of bias, inconsistency and imprecision).	Yes
**Interpretation**	10	Provide a general interpretation of the results and important implications.	Yes
**OTHER**			
**Funding**	11	Specify the primary source of funding for the review.	Yes
**Registration**	12	Provide the register name and registration number.	Yes

Table S4. Risk of bias based on modified Newcastle-Ottawa scale

Stars are given for low risk of bias in each category, up to a total of nine stars. Scoring details are in the footnotes.

Reference	Selection bias	Response bias	Follow-up bias	Misclassification bias	Outcome assessment	Outcome measurement	Bias in analysis	Total
	Is there evidence of selection bias, which refers to systematic differences between baseline characteristics of the groups that are compared?[a]	Is there evidence of response bias?[b]	Is there evidence of bias due to missing follow-up data?[c]	Is there risk of households not receiving the intervention being misclassified as having received it, or vice versa?[d]	Is there evidence of bias arising from how the outcome was assessed?[e]	Is there evidence of ascertainment bias?[f]	Is there evidence that analysis was not appropriately adjusted for clustering and/or confounding, if appropriate?[g]	Total number of stars (x/9 possible stars).
Clasen T, et al. Effectiveness of a rural sanitation programme on diarrhoea, soil-transmitted helminth infection, and child malnutrition in Odisha, India: a cluster-randomised trial. Lancet Glob Health. 2014.	*	* no, laboratory assessed and blinded	possible (86% of possible weeks are reported weeks)	* household-level interventions	**	*	** adjusted for clustering	8
Luby, S.P. et al.. Effects of water quality, sanitation, handwashing, and nutritional interventions on diarrhoea and child growth in rural Bangladesh: a cluster randomised controlled trial. The Lancet Global Health 2018	*	* no, laboratory assessed and blinded	* 94% complete FU	* household-level interventions	**	*	**	9

Table S4. Risk of bias based on modified Newcastle-Ottawa scale

Stars are given for low risk of bias in each category, up to a total of nine stars. Scoring details are in the footnotes.

Reference	Selection bias	Response bias	Follow-up bias	Misclassification bias	Outcome assessment	Outcome measurement	Bias in analysis	Total
Null, C. et al., Effects of water quality, sanitation, handwashing, and nutritional interventions on diarrhoea and child growth in rural Kenya: a cluster-randomised controlled trial. The Lancet Global Health 2018	*	* no, laboratory assessed and blinded	* <1% loss to FU	* household-level interventions	**	*	**	9
Reese, H. et al. Assessing longer-term effectiveness of a combined household-level piped water and sanitation intervention on child diarrhoea, acute respiratory infection, soil-transmitted helminth infection and nutritional status: a matched cohort study in rural Odisha, India. International journal of epidemiology 2019	selection bias is possible, as the study is not randomized and there are some baseline differences between intervention and control group	* no, laboratory assessed and blinded	substantial loss to FU	* household-level interventions	**	*	**	7

Table S4. Risk of bias based on modified Newcastle-Ottawa scale

Stars are given for low risk of bias in each category, up to a total of nine stars. Scoring details are in the footnotes.

Reference	Selection bias	Response bias	Follow-up bias	Misclassification bias	Outcome assessment	Outcome measurement	Bias in analysis	Total
Knee, J. et al. Effects of an urban sanitation intervention on childhood enteric infection and diarrhea in Maputo, Mozambique: A controlled before-and-after trial. eLife 2011	selection bias is possible, as the study is not randomized, but intervention and control groups were mostly balanced at baseline. Control households were more likely to have covered floors and higher quality walls and intervention groups had more people per household.	* no, laboratory assessed and blinded	substantial loss to FU	* household-level interventions	**	*	**	7

[a] RCTs receive 1 star, unless evidence of selection bias (e.g. randomisation procedures not followed). Meaningful differences between groups at baseline in RCTs receive 0 stars. Rates of declining to participate >10% receive 0 stars. Non- or quasi-randomised studies receive 0 stars.

[b] If intervention recipient was not blinded to intervention status, 0 stars.

[c] <10% receives 1 star, greater than or equal to 10% receives 0 stars.

[d] Interventions delivered at the household/individual level receive 1 star. Interventions delivered at the community level that missed a substantial, i.e. greater than or equal to 10%, proportion of the target population receive 0 stars, including when there is insufficient information to verify whether this is the case. Interventions with substantial risk of contamination (control households receiving intervention) receive 0 stars.

[e] Parent / person recall (=0 stars). Fieldworker assessed (=1 star). Physician/microbiologically assessed (=2 stars)

[f] If outcome measurement staff were not blinded to intervention status, 0 stars.

[g] Scoring is based on losing stars (max. 2). Individual RCTs with baseline balance on covariates are unlikely to require adjustment (=2 stars). Cluster-RCTs and non-randomised trials may require adjustment for clustering (-1 star if not done). RCTs or cRCTs may require adjustment for covariates, with justification (-1 star if not done). Non-randomised studies require adjustment for covariates (-1 star if not done), but also adequate justification for covariate selection (-1 star if not included), and there can be too few or too many covariates.

Table S5. Prevalence of pathogens by sample type tested in each study

Study	Sample	Target	Percent positive (n/N)	PR (95% CI)
Odagiri 2016	Source water	V. cholerae	31.7% (19/60)	0.73 (0.34, 1.57)
-	-	Adenovirus	8.3% (5/60)	0.25 (0.03, 2.19)
-	-	Rotavirus	23.3% (14/60)	0.75 (0.29, 1.93)
Boehm 2016	Stored water	Rotavirus	0.6% (3/493)	-
-	Child hand rinse	Rotavirus	6.1% (30/493)	-
-	House soil	Rotavirus	1.4% (7/496)	2.52 (0.51, 12.42)
Reese 2017	Source water	Shigella	10.7% (161/1499)	0.73 (0.46, 1.15)
-	-	V. cholerae	13% (36/276)	0.93 (0.46, 1.85)
-	Stored water	Shigella	10.1% (190/1874)	1.08 (0.77, 1.51)
-	-	V. cholerae	23.7% (100/422)	1.03 (0.66, 1.6)
Steinbaum 2019	House soil	Ascaris	13% (273/2107)	0.88 (0.68, 1.13)
-	-	Trichuris	6.9% (146/2107)	0.86 (0.6, 1.23)
Fuhrmeister 2020	Stored water	Pathogenic E. coli	38.6% (286/741)	1 (0.84, 1.19)
-	Child hand rinse	Pathogenic E. coli	34% (127/373)	-
-	-	Giardia	4.8% (15/311)	-
-	-	Norovirus	4.2% (14/337)	-
-	Mother's hand rinse	Pathogenic E. coli	24% (177/737)	-
-	-	Giardia	2.3% (14/602)	-
-	-	Norovirus	3.1% (21/684)	-
-	House soil	Pathogenic E. coli	61.3% (453/739)	0.94 (0.84, 1.06)
Capone 2021	Latrine soil	C. difficile	14.8% (13/88)	0.9 (0.32, 2.48)
-	-	Campylobacter	6.8% (6/88)	2.09 (0.4, 11.05)

Table S5. Prevalence of pathogens by sample type tested in each study

Study	Sample	Target	Percent positive (n/N)	PR (95% CI)
-	-	Pathogenic E. coli	56.8% (50/88)	0.89 (0.56, 1.42)
-	-	Salmonella	6.8% (6/88)	0.52 (0.1, 2.76)
-	-	Shigella	21.6% (19/88)	0.28 (0.1, 0.78)
-	-	V. cholerae	0% (0/88)	-
-	-	Yersinia	4.5% (4/88)	-
-	-	Ascaris	60.2% (53/88)	0.65 (0.41, 1.02)
-	-	Trichuris	17% (15/88)	0.92 (0.36, 2.33)
-	-	Cryptosporidium	8% (7/88)	0.78 (0.18, 3.36)
-	-	Entamoeba histolytica	1.1% (1/88)	-
-	-	Giardia	31.8% (28/88)	0.47 (0.21, 1.07)
-	-	Adenovirus	20.5% (18/88)	0.21 (0.06, 0.68)
-	-	Astrovirus	29.5% (26/88)	1.27 (0.67, 2.43)
-	-	Norovirus	2.3% (2/88)	-
-	-	Rotavirus	4.5% (4/88)	-
-	-	Sapovirus	0% (0/88)	-
Capone 2022 in prep	Flies	Campylobacter	1.2% (1/86)	-
-	-	Pathogenic E. coli	30.2% (26/86)	-
-	-	Shigella	2.3% (2/86)	-
-	-	V. cholerae	2.3% (2/86)	-
-	-	Ascaris	0% (0/86)	-
-	-	Trichuris	3.5% (3/86)	-
-	-	Giardia	4.7% (4/86)	-

Table S5. Prevalence of pathogens by sample type tested in each study

Study	Sample	Target	Percent positive (n/N)	PR (95% CI)
-	-	Adenovirus	4.7% (4/86)	-
-	-	Astrovirus	0% (0/86)	-
-	-	Norovirus	2.3% (2/86)	-
-	-	Pan enterovirus	0% (0/86)	-
-	-	Rotavirus	1.2% (1/86)	-
-	-	Sapovirus	0% (0/86)	-
Kwong 2021	House soil	Ascaris	62.1% (886/1426)	0.97 (0.87, 1.08)
-	-	Trichuris	56% (798/1426)	1.03 (0.91, 1.15)

Table S6. Prevalence of microbial source tracking markers by sample type tested in each study

Study	Sample	Target	Percent positive (n/N)	PR (95% CI)
Odagiri 2016	Source water	Animal (BacCow)	91.7% (55/60)	1.04 (0.89, 1.21)
-	-	Human (BacHum)	71.7% (43/60)	1.05 (0.76, 1.45)
Boehm 2016	Stored water	Avian (GFD)	9.3% (46/493)	0.71 (0.37, 1.36)
-	-	Ruminant (BacR)	21.9% (108/493)	0.62 (0.43, 0.9)
-	-	Human (HumM2)	0% (0/493)	-
-	Child hand rinse	Avian (GFD)	16.2% (80/493)	-
-	-	Ruminant (BacR)	54.2% (267/493)	-
-	-	Human (HumM2)	2.4% (12/493)	-
-	House soil	Avian (GFD)	33.3% (165/496)	0.98 (0.76, 1.27)
-	-	Ruminant (BacR)	66.7% (331/496)	0.98 (0.85, 1.12)
-	-	Human (HumM2)	8.9% (44/496)	0.94 (0.5, 1.75)
Fuhrmeister 2020	Stored water	Animal (BacCow)	68.5% (482/704)	0.97 (0.87, 1.08)
-	-	Human (HumM2)	2.6% (17/651)	0.44 (0.16, 1.23)
-	Child hand rinse	Animal (BacCow)	97.5% (356/365)	-
-	-	Human (HumM2)	21.9% (74/338)	-
-	Mother's hand rinse	Animal (BacCow)	96.7% (702/726)	-
-	-	Human (HumM2)	18.1% (118/651)	-
-	House soil	Animal (BacCow)	90.6% (572/631)	0.99 (0.94, 1.04)
-	-	Human (HumM2)	20.1% (127/631)	1.24 (0.91, 1.7)
Holcomb 2021	Source water	Avian (GFD)	0% (0/41)	-
-	-	Human (HF183)	2.4% (1/41)	-
-	-	Human (M. smithii)	0% (0/41)	-

Table S6. Prevalence of microbial source tracking markers by sample type tested in each study

Study	Sample	Target	Percent positive (n/N)	PR (95% CI)
-	Stored water	Avian (GFD)	1.1% (1/94)	-
-	-	Human (HF183)	14.9% (14/94)	1.72 (0.57, 5.18)
-	-	Human (M. smithii)	0% (0/94)	-
-	Latrine soil	Avian (GFD)	3.3% (2/60)	-
-	-	Human (HF183)	50% (30/60)	0.88 (0.51, 1.52)
-	-	Human (M. smithii)	45% (27/60)	0.74 (0.36, 1.55)
-	House soil	Avian (GFD)	3.6% (3/83)	-
-	-	Human (HF183)	42.2% (35/83)	0.81 (0.49, 1.34)
-	-	Human (M. smithii)	24.1% (20/83)	1.3 (0.62, 2.73)
Capone 2022 in prep	Flies	Animal (BacCow)	12.8% (11/86)	-
-	-	Dog (BacCan)	30.2% (26/86)	-
-	-	Human (BacHum)	72.1% (62/86)	-

Table S7.
Unadjusted and adjusted results by study, sample type, and aggregated variables for pathogen targets (any pathogen, any bacteria, any viruses, any protozoa, any STH).

Study	Target	Sample	Positive, Intervention	Negative, Intervention	Positive, Control	Negative, Control	Total observations	Unadjusted Prevalence Ratio	Unadjusted p-value	Adjusted Prevalence Ratio	Adjusted p-value
Capone 2022 in prep	Any pathogen	Any sample	7	13	20	17	57	PR=0.65 (95% CI: 0.33, 1.28)	0.21	PR=0.5 (95% CI: 0.21, 1.19)	0.12
Capone 2021	Any pathogen	Any sample	37	6	43	2	88	PR=0.9 (95% CI: 0.78, 1.03)	0.13	PR=0.9 (95% CI: 0.78, 1.03)	0.13
Fuhrmeister 2020	Any pathogen	Any sample	314	136	348	123	921	PR=0.94 (95% CI: 0.87, 1.02)	0.17	PR=0.94 (95% CI: 0.87, 1.02)	0.13
Steinbaum 2019	Any pathogen	Any sample	206	979	173	707	2,065	PR=0.88 (95% CI: 0.7, 1.11)	0.29	PR=0.87 (95% CI: 0.7, 1.09)	0.24
Reese 2017	Any pathogen	Any sample	185	792	238	825	2,040	PR=0.85 (95% CI: 0.66, 1.08)	0.18	PR=0.86 (95% CI: 0.68, 1.09)	0.21
Boehm 2016	Any pathogen	Any sample	19	229	15	234	497	PR=1.27 (95% CI: 0.6, 2.68)	0.53	PR=1.28 (95% CI: 0.62, 2.66)	0.5
Odagiri 2016	Any pathogen	Any sample	12	18	15	15	60	PR=0.8 (95% CI: 0.45, 1.42)	0.45		
Reese 2017	Any pathogen	Source water	68	588	122	747	1,525	PR=0.74 (95% CI: 0.49, 1.12)	0.15	PR=0.74 (95% CI: 0.5, 1.12)	0.16
Odagiri 2016	Any pathogen	Source water	12	18	15	15	60	PR=0.8 (95% CI: 0.45, 1.42)	0.45		
Fuhrmeister 2020	Any pathogen	Stored water	138	218	148	237	741	PR=1.01 (95% CI: 0.85, 1.2)	0.93	PR=1 (95% CI: 0.84, 1.19)	0.97
Reese 2017	Any pathogen	Stored water	134	786	147	860	1,927	PR=1 (95% CI: 0.75, 1.32)	0.99	PR=1.01 (95% CI: 0.77, 1.34)	0.94
Boehm 2016	Any pathogen	Stored water	2	243	1	245	491	Not estimated		Not estimated	
Kwong 2021	Any pathogen	House soil	363	125	687	221	1,396	PR=0.98 (95% CI: 0.91, 1.06)	0.67	PR=0.98 (95% CI: 0.91, 1.06)	0.68
Fuhrmeister 2020	Any pathogen	House soil	217	144	236	142	739	PR=0.96 (95% CI: 0.86, 1.08)	0.53	PR=0.94 (95% CI: 0.84, 1.06)	0.31

Table S7.

Unadjusted and adjusted results by study, sample type, and aggregated variables for pathogen targets (any pathogen, any bacteria, any viruses, any protozoa, any STH).

Study	Target	Sample	Positive, Intervention	Negative, Intervention	Positive, Control	Negative, Control	Total observations	Unadjusted Prevalence Ratio	Unadjusted p-value	Adjusted Prevalence Ratio	Adjusted p-value
Steinbaum 2019	Any pathogen	House soil	209	1,000	173	725	2,107	PR=0.9 (95% CI: 0.72, 1.13)	0.35	PR=0.89 (95% CI: 0.71, 1.11)	0.31
Boehm 2016	Any pathogen	House soil	5	242	2	247	496	PR=2.52 (95% CI: 0.51, 12.42)	0.26	PR=2.52 (95% CI: 0.51, 12.42)	0.26
Capone 2021	Any pathogen	Latrine soil	37	6	43	2	88	PR=0.9 (95% CI: 0.78, 1.03)	0.13	PR=0.9 (95% CI: 0.78, 1.03)	0.13
Capone 2022 in prep	Any pathogen		8	23	25	30	86	PR=0.57 (95% CI: 0.28, 1.15)	0.12	PR=0.37 (95% CI: 0.16, 0.85)	0.02
Fuhrmeister 2020	Any pathogen		75	113	72	116	376	PR=1.04 (95% CI: 0.8, 1.35)	0.76	PR=1.05 (95% CI: 0.81, 1.37)	0.69
Fuhrmeister 2020	Any pathogen		96	266	110	267	739	PR=0.91 (95% CI: 0.72, 1.15)	0.43	PR=0.92 (95% CI: 0.72, 1.16)	0.47
Boehm 2016	Any pathogen		16	231	14	232	493	PR=1.14 (95% CI: 0.52, 2.48)	0.75	PR=1.13 (95% CI: 0.52, 2.44)	0.76
Capone 2022 in prep	Any bacteria	Any sample	7	13	17	20	57	PR=0.76 (95% CI: 0.38, 1.54)	0.45	PR=0.6 (95% CI: 0.24, 1.46)	0.26
Capone 2021	Any bacteria	Any sample	28	15	35	10	88	PR=0.84 (95% CI: 0.64, 1.1)	0.2	PR=0.85 (95% CI: 0.65, 1.11)	0.24
Fuhrmeister 2020	Any bacteria	Any sample	306	144	340	131	921	PR=0.94 (95% CI: 0.86, 1.03)	0.18	PR=0.94 (95% CI: 0.86, 1.02)	0.14
Reese 2017	Any bacteria	Any sample	185	792	238	825	2,040	PR=0.85 (95% CI: 0.66, 1.08)	0.18	PR=0.86 (95% CI: 0.68, 1.09)	0.21
Odagiri 2016	Any bacteria	Any sample	8	22	11	19	60	PR=0.73 (95% CI: 0.34, 1.57)	0.42		
Reese 2017	Any bacteria	Source water	68	588	122	747	1,525	PR=0.74 (95% CI: 0.49, 1.12)	0.15	PR=0.74 (95% CI: 0.5, 1.12)	0.16
Odagiri 2016	Any bacteria	Source water	8	22	11	19	60	PR=0.73 (95% CI: 0.34, 1.57)	0.42		

Table S7.

Unadjusted and adjusted results by study, sample type, and aggregated variables for pathogen targets (any pathogen, any bacteria, any viruses, any protozoa, any STH).

Study	Target	Sample	Positive, Intervention	Negative, Intervention	Positive, Control	Negative, Control	Total observations	Unadjusted Prevalence Ratio	Unadjusted p-value	Adjusted Prevalence Ratio	Adjusted p-value
Fuhrmeister 2020	Any bacteria	Stored water	138	218	148	237	741	PR=1.01 (95% CI: 0.85, 1.2)	0.93	PR=1 (95% CI: 0.84, 1.19)	0.97
Reese 2017	Any bacteria	Stored water	134	786	147	860	1,927	PR=1 (95% CI: 0.75, 1.32)	0.99	PR=1.01 (95% CI: 0.77, 1.34)	0.94
Fuhrmeister 2020	Any bacteria	House soil	217	144	236	142	739	PR=0.96 (95% CI: 0.86, 1.08)	0.53	PR=0.94 (95% CI: 0.84, 1.06)	0.31
Capone 2021	Any bacteria	Latrine soil	28	15	35	10	88	PR=0.84 (95% CI: 0.64, 1.1)	0.2	PR=0.85 (95% CI: 0.65, 1.11)	0.24
Capone 2022 in prep	Any bacteria		8	23	21	34	86	PR=0.68 (95% CI: 0.32, 1.41)	0.3	PR=0.62 (95% CI: 0.28, 1.38)	0.24
Fuhrmeister 2020	Any bacteria		64	122	63	124	373	PR=1.02 (95% CI: 0.78, 1.35)	0.88	PR=1.02 (95% CI: 0.78, 1.35)	0.88
Fuhrmeister 2020	Any bacteria		81	281	96	279	737	PR=0.87 (95% CI: 0.68, 1.13)	0.3	PR=0.85 (95% CI: 0.67, 1.09)	0.2
Capone 2022 in prep	Any virus	Any sample	0	20	4	33	57	Not estimated		Not estimated	
Capone 2021	Any virus	Any sample	16	27	22	23	88	PR=0.76 (95% CI: 0.46, 1.25)	0.28	PR=0.63 (95% CI: 0.35, 1.14)	0.13
Fuhrmeister 2020	Any virus	Any sample	17	330	14	338	699	PR=1.23 (95% CI: 0.63, 2.4)	0.54	PR=1.22 (95% CI: 0.63, 2.34)	0.56
Boehm 2016	Any virus	Any sample	19	229	15	234	497	PR=1.27 (95% CI: 0.6, 2.68)	0.53	PR=1.28 (95% CI: 0.62, 2.66)	0.5
Odagiri 2016	Any virus	Any sample	7	23	10	20	60	PR=0.7 (95% CI: 0.3, 1.62)	0.4		
Odagiri 2016	Any virus	Source water	7	23	10	20	60	PR=0.7 (95% CI: 0.3, 1.62)	0.4		
Boehm 2016	Any virus	Stored water	2	243	1	245	491	Not estimated		Not estimated	

Table S7.

Unadjusted and adjusted results by study, sample type, and aggregated variables for pathogen targets (any pathogen, any bacteria, any viruses, any protozoa, any STH).

Study	Target	Sample	Positive, Intervention	Negative, Intervention	Positive, Control	Negative, Control	Total observations	Unadjusted Prevalence Ratio	Unadjusted p-value	Adjusted Prevalence Ratio	Adjusted p-value
Boehm 2016	Any virus	House soil	5	242	2	247	496	PR=2.52 (95% CI: 0.51, 12.42)	0.26	PR=2.52 (95% CI: 0.51, 12.42)	0.26
Capone 2021	Any virus	Latrine soil	16	27	22	23	88	PR=0.76 (95% CI: 0.46, 1.25)	0.28	PR=0.63 (95% CI: 0.35, 1.14)	0.13
Capone 2022 in prep	Any virus		0	31	5	50	86	PR=0 (95% CI: 0, 0)	0	PR=0 (95% CI: 0, 0)	0
Fuhrmeister 2020	Any virus		7	162	7	161	337	PR=0.99 (95% CI: 0.37, 2.69)	0.99	PR=0.99 (95% CI: 0.37, 2.69)	0.99
Fuhrmeister 2020	Any virus		11	331	10	332	684	PR=1.1 (95% CI: 0.47, 2.57)	0.83	PR=1.06 (95% CI: 0.45, 2.46)	0.9
Boehm 2016	Any virus		16	231	14	232	493	PR=1.14 (95% CI: 0.52, 2.48)	0.75	PR=1.13 (95% CI: 0.52, 2.44)	0.76
Capone 2022 in prep	Any protozoa	Any sample	0	20	3	34	57	Not estimated		Not estimated	
Capone 2021	Any protozoa	Any sample	15	28	19	26	88	PR=0.83 (95% CI: 0.48, 1.42)	0.49	PR=0.83 (95% CI: 0.48, 1.42)	0.49
Fuhrmeister 2020	Any protozoa	Any sample	12	293	16	291	612	PR=0.75 (95% CI: 0.35, 1.65)	0.48	PR=0.77 (95% CI: 0.35, 1.67)	0.5
Capone 2021	Any protozoa	Latrine soil	15	28	19	26	88	PR=0.83 (95% CI: 0.48, 1.42)	0.49	PR=0.83 (95% CI: 0.48, 1.42)	0.49
Capone 2022 in prep	Any protozoa		0	31	4	51	86	Not estimated		Not estimated	
Fuhrmeister 2020	Any protozoa		7	147	8	149	311	PR=0.89 (95% CI: 0.33, 2.38)	0.82	PR=0.89 (95% CI: 0.33, 2.38)	0.82
Fuhrmeister 2020	Any protozoa		5	296	9	292	602	PR=0.56 (95% CI: 0.14, 2.13)	0.39	PR=0.56 (95% CI: 0.14, 2.13)	0.39
Capone 2022 in prep	Any STH	Any sample	0	20	3	34	57	Not estimated		Not estimated	

Table S7.

Unadjusted and adjusted results by study, sample type, and aggregated variables for pathogen targets (any pathogen, any bacteria, any viruses, any protozoa, any STH).

Study	Target	Sample	Positive, Intervention	Negative, Intervention	Positive, Control	Negative, Control	Total observations	Unadjusted Prevalence Ratio	Unadjusted p-value	Adjusted Prevalence Ratio	Adjusted p-value
Capone 2021	Any STH	Any sample	20	23	34	11	88	PR=0.62 (95% CI: 0.43, 0.89)	0.01	PR=0.69 (95% CI: 0.45, 1.07)	0.1
Steinbaum 2019	Any STH	Any sample	206	979	173	707	2,065	PR=0.88 (95% CI: 0.7, 1.11)	0.29	PR=0.87 (95% CI: 0.7, 1.09)	0.24
Kwong 2021	Any STH	House soil	363	125	687	221	1,396	PR=0.98 (95% CI: 0.91, 1.06)	0.67	PR=0.98 (95% CI: 0.91, 1.06)	0.68
Steinbaum 2019	Any STH	House soil	209	1,000	173	725	2,107	PR=0.9 (95% CI: 0.72, 1.13)	0.35	PR=0.89 (95% CI: 0.71, 1.11)	0.31
Capone 2021	Any STH	Latrine soil	20	23	34	11	88	PR=0.62 (95% CI: 0.43, 0.89)	0.01	PR=0.69 (95% CI: 0.45, 1.07)	0.1
Capone 2022 in prep	Any STH		0	31	3	52	86	Not estimated		Not estimated	

Table S8.
Unadjusted and adjusted results by study, sample type, and aggregated variables for MST targets (any MST, any general MST, any human MST, any animal MST).

Study	Target	Sample	Positive, Intervention	Negative, Intervention	Positive, Control	Negative, Control	Total observations	Unadjusted Prevalence Ratio	Unadjusted p-value	Adjusted Prevalence Ratio	Adjusted p-value
Capone 2022 in prep	Any MST Marker	Any sample	20	0	32	5	57	PR=1.16 (95% CI: 1.02, 1.32)	0.03	PR=1.16 (95% CI: 1.02, 1.32)	0.03
Holcomb 2021	Any MST Marker	Any sample	41	28	44	17	130	PR=0.82 (95% CI: 0.62, 1.09)	0.18	PR=0.86 (95% CI: 0.65, 1.13)	0.27
Fuhrmeister 2020	Any MST Marker	Any sample	421	26	438	29	914	PR=1 (95% CI: 0.97, 1.04)	0.8	PR=1.01 (95% CI: 0.97, 1.04)	0.7
Boehm 2016	Any MST Marker	Any sample	220	28	222	27	497	PR=0.99 (95% CI: 0.93, 1.06)	0.88	PR=0.99 (95% CI: 0.93, 1.06)	0.76
Odagiri 2016	Any MST Marker	Any sample	30	0	28	2	60	Not estimated			
Holcomb 2021	Any MST Marker	Source water	1	21	0	19	41	Not estimated		Not estimated	
Odagiri 2016	Any MST Marker	Source water	30	0	28	2	60	Not estimated			
Holcomb 2021	Any MST Marker	Stored water	9	39	6	40	94	PR=1.44 (95% CI: 0.51, 4.08)	0.5	PR=1.44 (95% CI: 0.51, 4.08)	0.5
Fuhrmeister 2020	Any MST Marker	Stored water	230	119	256	119	724	PR=0.97 (95% CI: 0.87, 1.07)	0.52	PR=0.97 (95% CI: 0.88, 1.08)	0.63
Boehm 2016	Any MST Marker	Stored water	57	188	82	164	491	PR=0.7 (95% CI: 0.51, 0.96)	0.03	PR=0.69 (95% CI: 0.5, 0.95)	0.02
Holcomb 2021	Any MST Marker	House soil	21	18	26	18	83	PR=0.91 (95% CI: 0.6, 1.38)	0.66	PR=0.89 (95% CI: 0.62, 1.28)	0.54

Table S8.
Unadjusted and adjusted results by study, sample type, and aggregated variables for MST targets (any MST, any general MST, any human MST, any animal MST).

Study	Target	Sample	Positive, Intervention	Negative, Intervention	Positive, Control	Negative, Control	Total observations	Unadjusted Prevalence Ratio	Unadjusted p-value	Adjusted Prevalence Ratio	Adjusted p-value
Fuhrmeister 2020	Any MST Marker	House soil	283	38	297	36	654	PR=0.99 (95% CI: 0.93, 1.05)	0.7	PR=0.99 (95% CI: 0.93, 1.05)	0.66
Boehm 2016	Any MST Marker	House soil	180	67	187	62	496	PR=0.97 (95% CI: 0.87, 1.08)	0.59	PR=0.97 (95% CI: 0.87, 1.08)	0.58
Holcomb 2021	Any MST Marker	Latrine soil	21	9	22	8	60	PR=0.95 (95% CI: 0.69, 1.32)	0.78	PR=0.95 (95% CI: 0.69, 1.32)	0.78
Capone 2022 in prep	Any MST Marker		27	4	42	13	86	PR=1.14 (95% CI: 0.93, 1.39)	0.2	PR=1.14 (95% CI: 0.93, 1.39)	0.2
Fuhrmeister 2020	Any MST Marker		174	11	182	1	368	PR=0.95 (95% CI: 0.91, 0.98)	0.01	PR=0.95 (95% CI: 0.91, 0.98)	0.01
Fuhrmeister 2020	Any MST Marker		346	14	359	9	728	PR=0.99 (95% CI: 0.96, 1.01)	0.26	PR=0.99 (95% CI: 0.96, 1.01)	0.29
Boehm 2016	Any MST Marker		145	102	148	98	493	PR=0.98 (95% CI: 0.82, 1.16)	0.78	PR=0.97 (95% CI: 0.82, 1.15)	0.74
Capone 2022 in prep	Any human MST Marker	Any sample	17	3	30	7	57	PR=1.05 (95% CI: 0.82, 1.34)	0.71	PR=1.05 (95% CI: 0.82, 1.34)	0.71
Holcomb 2021	Any human MST Marker	Any sample	41	28	43	18	130	PR=0.84 (95% CI: 0.63, 1.12)	0.24	PR=0.89 (95% CI: 0.67, 1.18)	0.41
Fuhrmeister 2020	Any human MST Marker	Any sample	124	313	133	330	900	PR=0.99 (95% CI: 0.8, 1.22)	0.91	PR=1.01 (95% CI: 0.82, 1.25)	0.92
Boehm 2016	Any human MST Marker	Any sample	26	222	26	223	497	PR=1 (95% CI: 0.57, 1.75)	0.99	PR=1 (95% CI: 0.57, 1.76)	0.99

Table S8.

Unadjusted and adjusted results by study, sample type, and aggregated variables for MST targets (any MST, any general MST, any human MST, any animal MST).

Study	Target	Sample	Positive, Intervention	Negative, Intervention	Positive, Control	Negative, Control	Total observations	Unadjusted Prevalence Ratio	Unadjusted p-value	Adjusted Prevalence Ratio	Adjusted p-value
Odagiri 2016	Any human MST Marker	Any sample	22	8	21	9	60	PR=1.05 (95% CI: 0.76, 1.45)	0.78		
Holcomb 2021	Any human MST Marker	Source water	1	21	0	19	41	Not estimated		Not estimated	
Odagiri 2016	Any human MST Marker	Source water	22	8	21	9	60	PR=1.05 (95% CI: 0.76, 1.45)	0.78		
Holcomb 2021	Any human MST Marker	Stored water	9	39	5	41	94	PR=1.72 (95% CI: 0.57, 5.18)	0.33	PR=1.72 (95% CI: 0.57, 5.18)	0.33
Fuhrmeister 2020	Any human MST Marker	Stored water	5	310	12	324	651	PR=0.44 (95% CI: 0.16, 1.23)	0.12	PR=0.44 (95% CI: 0.16, 1.23)	0.12
Boehm 2016	Any human MST Marker	Stored water	0	245	0	246	491	Not estimated		Not estimated	
Holcomb 2021	Any human MST Marker	House soil	20	19	26	18	83	PR=0.87 (95% CI: 0.57, 1.32)	0.5	PR=0.86 (95% CI: 0.6, 1.24)	0.42
Fuhrmeister 2020	Any human MST Marker	House soil	68	243	59	261	631	PR=1.19 (95% CI: 0.87, 1.61)	0.28	PR=1.24 (95% CI: 0.91, 1.7)	0.18
Boehm 2016	Any human MST Marker	House soil	21	226	23	226	496	PR=0.92 (95% CI: 0.5, 1.71)	0.79	PR=0.94 (95% CI: 0.5, 1.75)	0.84
Holcomb 2021	Any human MST Marker	Latrine soil	21	9	22	8	60	PR=0.95 (95% CI: 0.69, 1.32)	0.78	PR=0.95 (95% CI: 0.69, 1.32)	0.78
Capone 2022 in prep	Any human MST Marker		24	7	38	17	86	PR=1.12 (95% CI: 0.83, 1.51)	0.46	PR=1.02 (95% CI: 0.75, 1.41)	0.88

Table S8.
Unadjusted and adjusted results by study, sample type, and aggregated variables for MST targets (any MST, any general MST, any human MST, any animal MST).

Study	Target	Sample	Positive, Intervention	Negative, Intervention	Positive, Control	Negative, Control	Total observations	Unadjusted Prevalence Ratio	Unadjusted p-value	Adjusted Prevalence Ratio	Adjusted p-value
Fuhrmeister 2020	Any human MST Marker		30	142	44	122	338	PR=0.66 (95% CI: 0.44, 0.99)	0.04	PR=0.72 (95% CI: 0.48, 1.07)	0.11
Fuhrmeister 2020	Any human MST Marker		58	268	60	265	651	PR=0.96 (95% CI: 0.68, 1.37)	0.84	PR=0.96 (95% CI: 0.68, 1.35)	0.82
Boehm 2016	Any human MST Marker		7	240	5	241	493	PR=1.39 (95% CI: 0.46, 4.2)	0.56	PR=1.39 (95% CI: 0.46, 4.2)	0.56
Capone 2022 in prep	Any animal MST Marker	Any sample	12	8	17	20	57	PR=1.31 (95% CI: 0.78, 2.17)	0.3	PR=1.2 (95% CI: 0.72, 1.99)	0.48
Holcomb 2021	Any animal MST Marker	Any sample	3	66	2	59	130	PR=1.33 (95% CI: 0.18, 9.59)	0.78	PR=1.33 (95% CI: 0.18, 9.59)	0.78
Fuhrmeister 2020	Any animal MST Marker	Any sample	419	26	437	28	910	PR=1 (95% CI: 0.97, 1.04)	0.91	PR=1 (95% CI: 0.97, 1.04)	0.8
Boehm 2016	Any animal MST Marker	Any sample	219	29	221	28	497	PR=0.99 (95% CI: 0.93, 1.06)	0.88	PR=0.99 (95% CI: 0.93, 1.06)	0.74
Odagiri 2016	Any animal MST Marker	Any sample	28	2	27	3	60	PR=1.04 (95% CI: 0.89, 1.21)	0.65		
Holcomb 2021	Any animal MST Marker	Source water	0	22	0	19	41	Not estimated		Not estimated	
Odagiri 2016	Any animal MST Marker	Source water	28	2	27	3	60	PR=1.04 (95% CI: 0.89, 1.21)	0.65		
Holcomb 2021	Any animal MST Marker	Stored water	0	48	1	45	94	Not estimated		Not estimated	

Table S8.
Unadjusted and adjusted results by study, sample type, and aggregated variables for MST targets (any MST, any general MST, any human MST, any animal MST).

Study	Target	Sample	Positive, Intervention	Negative, Intervention	Positive, Control	Negative, Control	Total observations	Unadjusted Prevalence Ratio	Unadjusted p-value	Adjusted Prevalence Ratio	Adjusted p-value
Fuhrmeister 2020	Any animal MST Marker	Stored water	229	113	253	109	704	PR=0.96 (95% CI: 0.86, 1.07)	0.43	PR=0.97 (95% CI: 0.87, 1.08)	0.57
Boehm 2016	Any animal MST Marker	Stored water	57	188	82	164	491	PR=0.7 (95% CI: 0.51, 0.96)	0.03	PR=0.69 (95% CI: 0.5, 0.95)	0.02
Holcomb 2021	Any animal MST Marker	House soil	2	37	1	43	83	Not estimated		Not estimated	
Fuhrmeister 2020	Any animal MST Marker	House soil	281	30	291	29	631	PR=0.99 (95% CI: 0.94, 1.05)	0.82	PR=0.99 (95% CI: 0.94, 1.04)	0.72
Boehm 2016	Any animal MST Marker	House soil	178	69	186	63	496	PR=0.96 (95% CI: 0.86, 1.08)	0.53	PR=0.96 (95% CI: 0.86, 1.08)	0.51
Holcomb 2021	Any animal MST Marker	Latrine soil	2	28	0	30	60	Not estimated		Not estimated	
Capone 2022 in prep	Any animal MST Marker		12	19	18	37	86	PR=1.18 (95% CI: 0.7, 2)	0.53	PR=1.33 (95% CI: 0.62, 2.86)	0.47
Fuhrmeister 2020	Any animal MST Marker		174	8	182	1	365	PR=0.96 (95% CI: 0.93, 1)	0.03	PR=0.96 (95% CI: 0.93, 1)	0.03
Fuhrmeister 2020	Any animal MST Marker		344	15	358	9	726	PR=0.98 (95% CI: 0.96, 1.01)	0.17	PR=0.98 (95% CI: 0.96, 1.01)	0.19
Boehm 2016	Any animal MST Marker		144	103	147	99	493	PR=0.98 (95% CI: 0.82, 1.16)	0.78	PR=0.97 (95% CI: 0.82, 1.15)	0.7

Table S9.

Baseline covariates by study. Note that Odigari et al. 2016 is not included as data shared from this study were from village water sources and did not have associated covariates from individual households; therefore all estimates for this study are unadjusted.

.	Boehm 2016	Reese 2017	Steinbaum 2019	Fuhrmeister 2020	Holcomb 2021	Capone 2021	Capone 2022 in prep.	Kwong 2021
Number of rooms in the household								
Household wealth								
Low	125 (25.2%)	28 (11.6%)	861 (40.9%)	153 (25.6%)	45 (27.6%)	22 (25.0%)	14 (24.6%)	355 (25.4%)
Medium-low	124 (24.9%)	51 (21.1%)	439 (20.8%)	145 (24.3%)	46 (28.2%)	23 (26.1%)	18 (31.6%)	343 (24.6%)
Medium-high	125 (25.2%)	39 (16.1%)	402 (19.1%)	147 (24.6%)	35 (21.5%)	22 (25.0%)	12 (21.1%)	351 (25.1%)
High	123 (24.7%)	65 (26.9%)	403 (19.1%)	152 (25.5%)	37 (22.7%)	21 (23.9%)	13 (22.8%)	347 (24.9%)
Missing	0 (0%)	59 (24.4%)	2 (0.1%)	0 (0%)	0 (0%)	0 (0%)	0 (0%)	0 (0%)
Number of people in the household								
<5	271 (54.5%)	17 (7.0%)	612 (29.0%)	335 (56.1%)	38 (23.3%)	0 (0%)	0 (0%)	783 (56.1%)
5-8	199 (40.0%)	171 (70.7%)	1149 (54.5%)	224 (37.5%)	44 (27.0%)	7 (8.0%)	3 (5.3%)	528 (37.8%)
5-8	199 (40.0%)	171 (70.7%)	1149 (54.5%)	224 (37.5%)	44 (27.0%)	7 (8.0%)	3 (5.3%)	528 (37.8%)
>8	27 (5.4%)	54 (22.3%)	245 (11.6%)	38 (6.4%)	81 (49.7%)	81 (92.0%)	54 (94.7%)	85 (6.1%)
Missing	0 (0%)	0 (0%)	101 (4.8%)	0 (0%)	0 (0%)	0 (0%)	0 (0%)	0 (0%)
Number of rooms in the household								
1-2	0 (0%)	0 (0%)	0 (0%)	0 (0%)	98 (60.1%)	61 (69.3%)	41 (71.9%)	0 (0%)

Table S9.

Baseline covariates by study. Note that Odigari et al. 2016 is not included as data shared from this study were from village water sources and did not have associated covariates from individual households; therefore all estimates for this study are unadjusted.

.	Boehm 2016	Reese 2017	Steinbaum 2019	Fuhrmeister 2020	Holcomb 2021	Capone 2021	Capone 2022 in prep.	Kwong 2021
>3	0 (0%)	0 (0%)	0 (0%)	0 (0%)	65 (39.9%)	27 (30.7%)	16 (28.1%)	0 (0%)
Missing	497 (100%)	242 (100%)	2107 (100%)	597 (100%)	0 (0%)	0 (0%)	0 (0%)	1396 (100%)
Improved roof								
0	8 (1.6%)	0 (0%)	693 (32.9%)	8 (1.3%)	0 (0%)	0 (0%)	0 (0%)	23 (1.6%)
1	489 (98.4%)	0 (0%)	1414 (67.1%)	589 (98.7%)	0 (0%)	0 (0%)	0 (0%)	1373 (98.4%)
Missing	0 (0%)	242 (100%)	0 (0%)	0 (0%)	163 (100%)	88 (100%)	57 (100%)	0 (0%)
Father in agriculture								
0	332 (66.8%)	126 (52.1%)	0 (0%)	419 (70.2%)	0 (0%)	0 (0%)	0 (0%)	952 (68.2%)
1	165 (33.2%)	89 (36.8%)	0 (0%)	178 (29.8%)	0 (0%)	0 (0%)	0 (0%)	444 (31.8%)
Missing	0 (0%)	27 (11.2%)	2107 (100%)	0 (0%)	163 (100%)	88 (100%)	57 (100%)	0 (0%)
Land owned								
0	0 (0%)	97 (40.1%)	0 (0%)	0 (0%)	0 (0%)	0 (0%)	0 (0%)	0 (0%)
1	0 (0%)	117 (48.3%)	0 (0%)	0 (0%)	0 (0%)	0 (0%)	0 (0%)	0 (0%)
Missing	497 (100%)	28 (11.6%)	2107 (100%)	597 (100%)	163 (100%)	88 (100%)	57 (100%)	1396 (100%)
Acres of land owned								
Mean (SD)	0.110 (0.128)	Missing	Missing	0.150 (0.206)	Missing	Missing	Missing	0.142 (0.212)

Table S9.

Baseline covariates by study. Note that Odigari et al. 2016 is not included as data shared from this study were from village water sources and did not have associated covariates from individual households; therefore all estimates for this study are unadjusted.

.	Boehm 2016	Reese 2017	Steinbaum 2019	Fuhrmeister 2020	Holcomb 2021	Capone 2021	Capone 2022 in prep.	Kwong 2021
Median [Min, Max]	0.0700 [0.0100, 1.23]	Missing	Missing	0.0800 [0.0100, 2.10]	Missing	Missing	Missing	0.0800 [0.0100, 3.15]
Missing	13 (2.6%)	242 (100%)	2107 (100%)	21 (3.5%)	163 (100%)	88 (100%)	57 (100%)	62 (4.4%)
Maternal education								
No education	85 (17.1%)	0 (0%)	0 (0%)	86 (14.4%)	6 (3.7%)	0 (0%)	0 (0%)	207 (14.8%)
Incomplete Primary	0 (0%)	83 (34.3%)	1095 (52.0%)	0 (0%)	38 (23.3%)	0 (0%)	0 (0%)	0 (0%)
Primary	180 (36.2%)	30 (12.4%)	511 (24.3%)	183 (30.7%)	14 (8.6%)	0 (0%)	0 (0%)	449 (32.2%)
Secondary	232 (46.7%)	70 (28.9%)	499 (23.7%)	328 (54.9%)	41 (25.2%)	0 (0%)	0 (0%)	740 (53.0%)
More than secondary	0 (0%)	11 (4.5%)	0 (0%)	0 (0%)	0 (0%)	0 (0%)	0 (0%)	0 (0%)
Missing	0 (0%)	48 (19.8%)	2 (0.1%)	0 (0%)	64 (39.3%)	88 (100%)	57 (100%)	0 (0%)
Maternal age								
Mean (SD)	23.7 (5.18)	Missing	26.4 (6.32)	23.7 (5.08)	Missing	Missing	Missing	24.0 (5.03)
Median [Min, Max]	23.0 [15.0, 42.0]	Missing	25.5 [14.9, 47.9]	23.0 [15.0, 41.0]	Missing	Missing	Missing	24.0 [15.0, 43.0]
Missing	0 (0%)	242 (100%)	11 (0.5%)	0 (0%)	163 (100%)	88 (100%)	57 (100%)	2 (0.1%)
Improved wall								
0	78 (15.7%)	0 (0%)	2019 (95.8%)	197 (33.0%)	41 (25.2%)	16 (18.2%)	10 (17.5%)	369 (26.4%)
1	419 (84.3%)	0 (0%)	88 (4.2%)	400 (67.0%)	122 (74.8%)	72 (81.8%)	47 (82.5%)	1027 (73.6%)

Table S9.

Baseline covariates by study. Note that Odigari et al. 2016 is not included as data shared from this study were from village water sources and did not have associated covariates from individual households; therefore all estimates for this study are unadjusted.

.	Boehm 2016	Reese 2017	Steinbaum 2019	Fuhrmeister 2020	Holcomb 2021	Capone 2021	Capone 2022 in prep.	Kwong 2021
Missing	0 (0%)	242 (100%)	0 (0%)	0 (0%)	0 (0%)	0 (0%)	0 (0%)	0 (0%)
Improved floor								
0	461 (92.8%)	0 (0%)	1999 (94.9%)	524 (87.8%)	4 (2.5%)	1 (1.1%)	1 (1.8%)	1253 (89.8%)
1	36 (7.2%)	0 (0%)	108 (5.1%)	73 (12.2%)	159 (97.5%)	87 (98.9%)	56 (98.2%)	143 (10.2%)
Missing	0 (0%)	242 (100%)	0 (0%)	0 (0%)	0 (0%)	0 (0%)	0 (0%)	0 (0%)
Electricity								
0	234 (47.1%)	34 (14.0%)	1958 (92.9%)	246 (41.2%)	3 (1.8%)	4 (4.5%)	2 (3.5%)	584 (41.8%)
1	263 (52.9%)	202 (83.5%)	147 (7.0%)	351 (58.8%)	160 (98.2%)	84 (95.5%)	55 (96.5%)	812 (58.2%)
Missing	0 (0%)	6 (2.5%)	2 (0.1%)	0 (0%)	0 (0%)	0 (0%)	0 (0%)	0 (0%)
Animal ownership								
Mean (SD)	0.956 (0.206)	0.423 (0.495)	0.899 (0.302)	0.955 (0.208)	0.896 (0.307)	0.966 (0.183)	0.982 (0.132)	0.968 (0.177)
Median [Min, Max]	1.00 [0, 1.00]	0 [0, 1.00]	1.00 [0, 1.00]	1.00 [0, 1.00]	1.00 [0, 1.00]	1.00 [0, 1.00]	1.00 [0, 1.00]	1.00 [0, 1.00]
Missing	1 (0.2%)	29 (12.0%)	0 (0%)	1 (0.2%)	0 (0%)	0 (0%)	0 (0%)	1 (0.1%)

99
